# Rheological characterisation of clay-cement sealing suspensions with fractionated coal fly ashes

**DOI:** 10.1038/s41598-025-30793-w

**Published:** 2026-01-22

**Authors:** Jurij Delihowski, Piotr Izak

**Affiliations:** Faculty of Materials Science and Ceramics, AGH University of Science and Technology, Cracow, Poland

**Keywords:** Coal fly ash, Particle size fractions, Clay-cement suspensions, Sealing suspensions, Rheology, Energy science and technology, Engineering, Environmental sciences, Materials science

## Abstract

This study investigates the rheological behaviour of clay–cement sealing suspensions modified with dry size-fractionated coal fly ashes of contrasting chemical composition. High-calcium S1 ashes and siliceous S2 ashes were separated into ultrafine (< 10 μm), fine (5–20 μm), and middle (20–100 μm) fractions to isolate size-dependent reactivity. Rotational and oscillatory rheometry demonstrate that Ca-rich fractions induce rapid structuration: at 20wt% ash addition, the Bingham yield stress rises from ~ 20–30 Pa in reference suspensions to > 150 Pa, and exceeds 400 Pa at 30wt% S1 loading, accompanied by steep increases in storage modulus (G′ up to 10⁷–10⁸Pa for ultrafine fractions). In contrast, S2 ashes disperse the clay–cement network, lowering yield stress to ~ 15–60 Pa even at 40 wt% replacement and maintaining moderate viscoelastic stiffness (G′~10⁴–10⁵Pa). Particle fineness amplifies these trends: S1.UF promotes early hydration products precipitation and slip-layer formation, whereas S2.UF preserves fluidity. Sodium-silicate activation introduces a distinct threshold—at 1.0–1.5wt% Na₂SiO₃ the S1 systems undergo a sol–gel transition, with τ₀ surging above 200 Pa, while S2 systems respond gradually. These results quantitatively shows how ash chemistry and granulometry govern early yield stress, viscosity, and G′/G″ ratios, and identify composition windows for either rapid sealing (Ca-rich, fine fractions) or long-distance pumpability (Si-rich, fine fractions). The work demonstrates that fractionated fly ash provides an effective design tool for tailoring rheology of low-cement, sustainable sealing slurries for hydraulic and geotechnical applications.

## Introduction

 Clay-cement sealing suspensions represent a critical engineering solution in hydraulic infrastructure, environmental protection, and geotechnical engineering^1,2^. These specialized mixtures combine the plasticity and low permeability of clay materials with the binding properties of cement to create barriers that effectively control water flow and pollutant migration in soil and rock environments^3,4^. Their application is particularly vital in flood protection systems, waste containment facilities, dam construction, and particularly in cut-off walls, levee remediation, foundation barriers and environmental containment structures remediation of contaminated sites where preventing water migration is essential^5,6^. This balance is governed by the interplay between the colloidal clay framework, cement hydration, and the evolving pore-solution chemistry. Numerous studies have described the rheological signatures of cement–bentonite systems, including yield stress evolution, shear-thinning behaviour, and thixotropic rebuilding, all of which control pumpability, trench stability and early mechanical integrity.

For example, cement–bentonite cutoff walls in levees and dams aim for hydraulic conductivities on the order of 10^–8^–10^–9^m/s, while simultaneously developing compressive strengths of several MPa through cement hydration. In practice, grout must penetrate several meters to form a continuous wall (typically 5–10 m deep or more) and infiltrate fine fissures under modest pressure. Correspondingly, field workability targets initial yield stresses of only a few pascals, ensuring that pumps can deliver the slurry efficiently^7–9^. Bleeding—defined as water–solid separation—is maintained below 5%, and for well designed slurries through proper mix design and use of stabilizing admixtures should be less then 1%^4,7,10,11^.

The rheological properties of these suspensions are paramount to their performance across all stages. During injection or placement, the suspension must maintain sufficient fluidity to penetrate target zones while simultaneously exhibiting adequate stability against segregation and eventual water flow blockage^12,13^. The choice of rheological model (Bingham, Herschel–Bulkley, etc.) depends on the specific formulation^14–16^. Immediately after mixing, cement–bentonite slurries typically exhibit yield stresses of 10–100 Pa and plastic viscosities of 0.05–0.4 Pa·s, with both values increasing as bentonite content rises and water/binder ratio decreases^4,16,17^. These rheological parameters correspond well to the observed flow spreads (~ 240 mm) and low bleeding^18,19^.

After placement, controlled structuration kinetics are necessary to ensure proper setting and development of mechanical properties without premature stiffening^20–24^. The complex rheological behavior of clay-cement systems stems from the interaction between the clay’s colloidal properties and cement’s progressive hydration reactions, creating time-dependent viscoelastic responses that significantly influence both application parameters and final performance characteristics^25–30^.

Despite their widespread utiity^4,8,31^, these suspensions often face sustainability challenges due to the high cement content typically required. Cement manufacture is energy-intensive (≈ 0.9 kg CO_2_ per kg cement) and expensive, so sealing suspensions with very high cement dosages raise economic and environmental concerns^32–35^. The incorporation of supplementary cementitious materials (SCM) has emerged as a promising approach to address these limitations^36–40^.

Ground-granulated blast-furnace slag has been reported improving rheology, accelerate solidification, enhance seepage resistance, increase compressive strain and reducing hydraulic conductivity^41–44^. Highly reactive pozzolans—such as silica fume, metakaolin, or rice husk ash—have the opposite effect on rheology, increasing yield stress and thixotropy, which enhances segregation resistance but requires higher water or superplasticizer dosage^45–49^. Fiber reinforcement, although not an SCM, can significantly increase crack resistance and fracture toughness under both static and dynamic loading of cured materials, while improving rheological behavior of fresh pastes^10,50–54^.

The reactivity of many SCMs can be substantially enhanced through alkali activation or geopolymerisation, enabling their use as primary binders rather than passive replacements^55–60^. Alkali-activated fly ash systems form N–A–S–H gels with Si/Al ratios typically between 1.5 and 2.5, while slag-rich blends generate C–A–S–H gels with higher Ca/Si ratios of 0.8–1.4, and are substantially denser than the C–S–H (Ca/Si ≈ 1.7–2.0) produced during ordinary Portland cement hydration^57,60^. Hybrid alkali-activated systems—such as slag–fly ash blends—simultaneously develop C–(N)–A–S–H networks, resulting in accelerated gel precipitation and rapid percolation of the solid skeleton. Rheologically, these reactions manifest as sharp increases in yield stress immediately after mixing within the first hour, depending on activator concentration and the slag/FA ratio^61–63^.

However, fly ash, a by-product of coal combustion in power plants, represents both an environmental burden when improperly disposed of and a valuable resource when effectively utilized^64–67^. Its integration into clay-cement systems can potentially change cement content requirement, alter rheological performance, and improve key engineering properties such as permeability and mechanical strength^68–74^. Its spherical particles lower yield stress and plastic viscosity (“ball-bearing” effect), improving pumpability and stability while moderating early heat evolution. In hardened states, FA refines pore structure and enhances chemical, thermal and mechanical resistance, making FA one of the most adaptable SCMs for cut-off wall suspensions^75–80^.

The wide variability in fly ash composition and physical characteristics significantly influences its behaviour in cementitious mixtures^70,81–88^. The heterogeneity of fly ash particles, which differ in size, morphology, and chemical composition, creates challenges in predicting and controlling the rheological and hardening behavior of modified suspensions^6,69,89,90^. This variability is particularly evident when comparing high-calcium fly ashes derived from lignite combustion with siliceous fly ashes from bituminous coal sources, as their reactive phases and interaction mechanisms with clay-cement matrices differ substantially^76,86,91–97^. Class F (bituminous, siliceous) ashes usually contain only a few percent CaO by mass, whereas Class C (subbituminous/lignite, calcareous) ashes can have 25–30% CaO or more. High-Ca ashes contain phases (free lime, belite, ye’elimite, etc.) that hydrate rapidly and even form ettringite without gypsum. Siliceous ashes are primarily pozzolanic^64,65^. This compositional difference strongly affects final product parameters. Additional pretreatment of fly ash—such as chemical or mechanical activation, thermal treatment, or physical processing through fractionation and separation—offers a promising route to homogenize its properties and isolate fractions with more uniform characteristics, thereby improving its overall applicability in engineering and binder systems^98–104^.

From a circular economy perspective, the incorporation of both coal combustion by-products (such as fly ash) and coal mining by-products (clay materials) into engineered systems directly addresses the dual challenge of waste valorization and resource efficiency. Research demonstrates that utilizing these industrial by-products not only diverts significant waste streams from landfills and storage facilities but also enables the production of valuable construction materials, such as aggregates, bricks, and cementitious binders, thereby reducing reliance on Portland cement and natural aggregates^105–108^. This approach aligns with the principles of the circular economy, which emphasize the reduction, reuse, and recycling (3R) of materials to minimize environmental impact and maximize resource utility^109–112^.

Through systematic fly ahs particle size fractionation, the research reveals how different ash fractions can distinctly influence suspension rheology^113–116^. This expanding the toolkit for formulating sealing applications with tailored short-term flow properties^13,117,118^. This study therefore tackles a dual waste-utilization challenge: fractionated fly ash and waste-derived clay. While we demonstrate rheological feasibility, a full life-cycle or cost analysis is outside the present scope and is flagged as future work.

## Raw materials characterization

### Coal fly Ash

The investigation utilized two contrasting coal combustion residues from electrical generation facilities in Poland:

- S1.O, Ca-enriched ash obtained from brown coal processing at the Belchatow facility.
- S2.O, Si-rich ash from hard coal combustion at the Krakow heat-generation plant.

These ashes underwent aerodynamic classification and screen separation to yield following distinct granulometric categories:

- Ultrafine component (UF): particulates below 10 μm.
- Fine component (F): particulates predominantly within 5–20 μm range.
- Intermediate component (M): particulates spanning approximately 20–100 μm, separated through a 100 μm mesh screen following initial aerodynamic processing.

### Fly Ash fractions characteristic

**Fig. 1:**
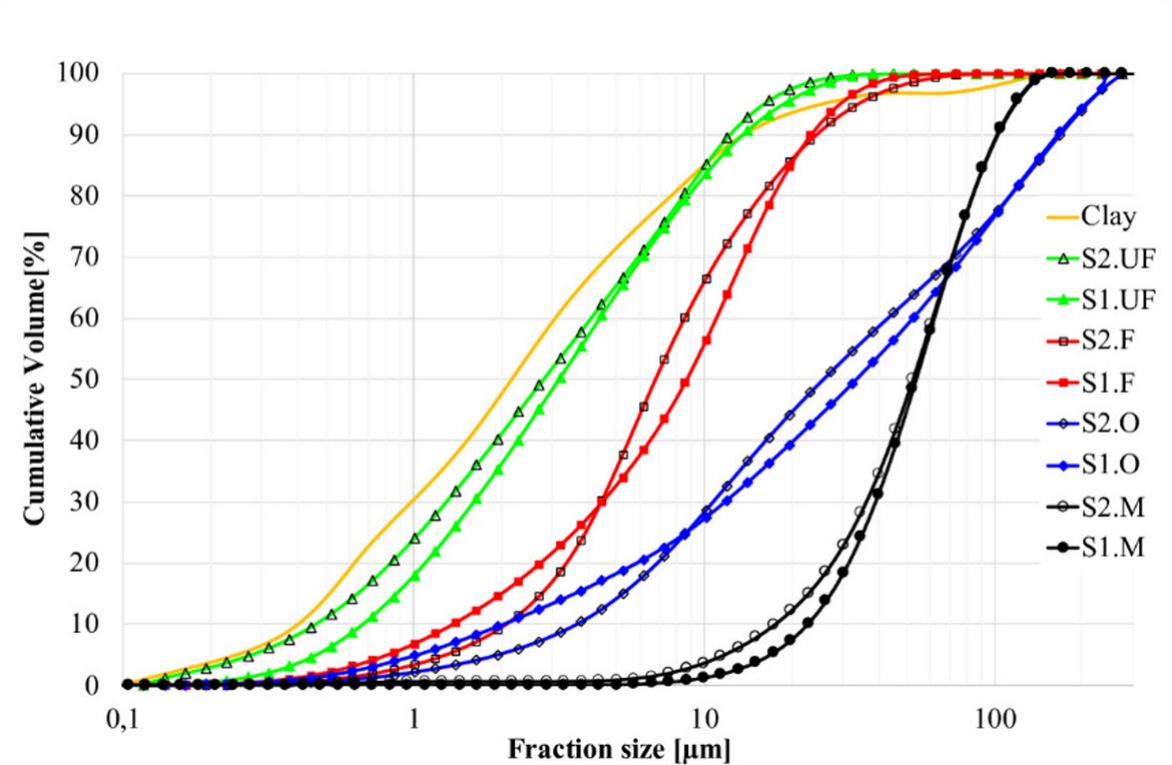
Granulometric distribution of obtained fly ash fractions and clay.

**Fig. 2:**
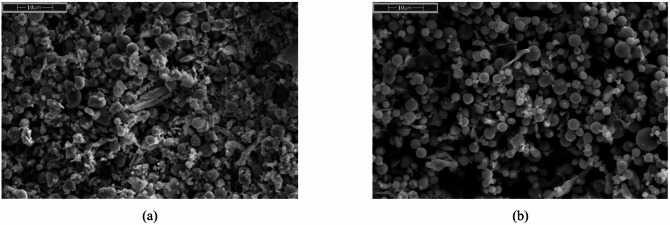
SEM images of fly ash fractions: (**a**) S1.UF, (**b**) S2.UF.

Dry aerodynamic classification combined with sieving yielded four distinct fly ash size fractions: *Ultra-fine (UF)* < 10 μm, *Fine (F)* ~ 5–20 μm, *Middle (M)* ~ 20–100 μm, and a remaining coarse fraction (the coarse fraction was excluded due to its low reactivity and tendency to cause sedimentation). Particle size distributions is presented at Fig. 1. SEM images of ultra fine (UF) fractions of both S1 and S2 ashes are presented at Fig. 2.

**Fig. 3:**
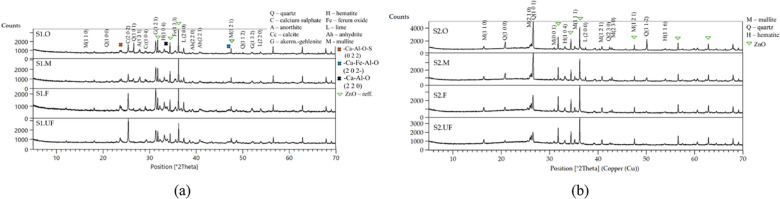
Mineral composition of: (**a**) S1 and (**b**) S2 ash fractions.

In this ashes, the chemical composition (Table 1) shifts with particle size. In the high-calcium ash (S1), finer fractions concentrate significantly higher CaO (up to ~ 37.8%) and SO₃ (~ 6.4%) than the middle fraction (CaO ~ 24.7%, SO₃ ~0.2%). Conversely, the siliceous ash (S2) is overall Si-rich (48–51% SiO₂) and low in CaO (4–7%), with alumina content and alkali oxides (K₂O, Na₂O) increase for finer fractions^119–122^. These trends align with prior studies on fractionated fly ashes of authors^123^. In this studies, the finer S1 fractions have showed the highest specific surface area (~ 15–18 m²/g) and density. In contrast, S2 fractions exhibit overall lower surface areas compared to S1 ashes (≤ 7.5 m²/g for S2.UF) and contain substantial loss-on-ignition (~ 13% LOI in fine/UF fractions) linked to unburned carbon^124^ XRD analysis (Fig. 3; Tables 2 and 3) confirms that finer S1 fractions are enriched in calcium-bearing phases capable of rapid hydration, whereas hard, quartz-rich grains content rises with particle size^102,125–128^. This observed distribution means that finer particles carry higher level of network-modifying cations (Ca²⁺, Mg²⁺, Na⁺, K⁺) in their amorphous (Si-Al) structure, making them readily dissolvable, while larger grains retain stable, silica-rich structure with limited early reactivity in watery solution^129–131^. These compositional gradients provide a foundation for interpreting the rheological behavior of the clay–cement–ash suspensions in subsequent sections.

It should be noted that the dry aerodynamic classifiers, as incorporated in this study, cannot achieve perfectly sharp cut-points, so the “coarse“ fraction still contains a minor tailings of fine particles and the “fine” fraction retains trace amounts of coarser grains. The resulting granulometric profiles (Fig. 1) and chemical compositions (Table 1) demonstrate clear differentiation between fractions, enabling systematic evaluation of size-dependent properties in subsequent rheological, structure-forming and mechanical analyses. Density and surface area of ash fractions are presented at Table 4.

**Table 1:** Chemical composition of S1 and S2 fly Ash fractions.

Chemical composition (%)								
	S1.O	S1.M	S1.F	S1.UF	S2.O	S2.M	S2.F	S2.UF
**SiO**_**2**_	**30.9**	**28.8**	**21.1**	**16.2**	**54.2**	**51.3**	**49.7**	**48.0**
**Al**_**2**_ **O**_**3**_	**30.1**	**28.8**	**26.0**	**24.1**	**24.5**	**23.0**	**29.5**	**30.1**
**CaO**	**24.7**	**25.4**	**33.9**	**37.8**	**5.1**	**6.6**	**4.3**	**4.2**
SO_3_	0.2	3.4	4.7	6.4	0.4	0.4	1.0	1.3
Fe_2_O_3_	9.5	10.7	10.6	10.4	8.3	9.1	6.1	5.9
MgO	1.1	1.0	1.3	1.5	3.4	4.2	2.9	2.5
TiO_2_	0.7	0.7	0.6	0.6	1.0	0.9	1.3	1.3
P_2_O_5_	0.6	0.6	0.5	0.6	0.3	0.3	0.7	0.9
K_2_O	0.2	0.1	0.1	0.2	2.6	2.6	3.3	3.2
Na_2_O	-	-	-	-	1.0	1.5	2.3	2.4
Total Ʃ	98.0	99.5	98.8	97.8	100.8	99.9	101.1	99.8
LOI (%)	1.7	0.9	1.7	1.8	11.2	7.7	13.3	13.3

**Table 2:** Quantified mineral composition of S1 ashes.

[%]	S1.O	S1.M	S1.F	S1.UF
Gehlenite	10.1	13.2	17.6	8.4
Quartz	2.5	1.3	-	-
Hematite	6.7	3.6	5.1	5.3
Anorthite	5.0	6.5	-	-
Anhydrite	4.5	-	-	15.8
Cristobalite	-	14.1	-	-
Lime	-	-	-	2.1
CCaA ^1^	1.3	8.7	20.8	8.4
Amorph.	69.7	52.3	55.5	56.7

^1^Complex calcium alumo-silicate compounds.

**Table 3:** Quantified mineralogical composition of S2 ashes.

[%]	S2.O	S2.M	S2.F	S2.UF
Mullite	20.2	22.2	18.1	19.7
Quartz	17.2	15.2	6.0	5.9
Hematite	1.4	2.6	0.7	0.8
Lime	0.3	1.2	-	-
Amorph.	62.1	58.5	74.4	73.3

**Table 4:** Density and surface area of obtained Ash fractions.

	Dencity	Surf. area		Dencity	Surf. area
	g/cm^3^	m^2^/g		g/cm^3^	m^2^/g
S1.O	2.82	11.37	S2.O	2.31	6.27
S1.M	2.64	13.22	S2.M	2.27	3.81
S1.F	3.01	15.39	S2.F	2.52	6.71
S1.UF	3.07	17.81	S2.UF	2.67	7.45

### Cement

The hydraulic component CEM I 42.5 R manufactured at the “Odra” S.A. production facility (Opole, Poland), containing Portland clinker (95–100%) supplemented with minor constituents including calcium sulfate (0–5%), composition presented in Table 5. The specific gravity (density of solid particles) is ≈ 3.10 g/cm³.

**Table 5:** Chemical composition of cement CEM I 42,5 R Odra Opole.

Chemical composition (%)					
SiO_2_	Al_2_O_3_	Fe_2_O_3_	CaO	MgO	Na_2_O
19.8	6.2	2.6	63	1.4	0.6

### Clay

The cohesive element consisted of mineral matter associated with lignite extraction at the Bełchatów excavation site. Mineralogical examination confirmed this material primarily contains beidellite and silica phases, chemical composition presented at Table 6.

**Table 6:** Chemical composition of clay Belchatow.

Chemical composition (%)										
**SiO**_**2**_	**Al**_**2**_ **O**_**3**_	**CaO**	**MgO**	**Fe**_**2**_ **O**_**3**_	**MnO**	**Na**_**2**_ **O**_**x**_	**K**_**2**_ **O**	**TiO**_**2**_	**H**_**2**_ **O** ^**-**^	**LOI**
58.65	14.55	4.75	0.72	3.46	0.72	1.02	1.05	0.42	5.52	9.72

### Clay source

The beidellite-rich clay used in this study originates from the Bełchatów lignite deposit (central Poland), which is known for its extensive horizons of dioctahedral smectite–beidellite clays formed through the weathering of volcanic tuffs and redeposition in Neogene sediments. These materials are characterized by a high content of smectitic phases (≈ 50–60%), accompanied by kaolinite (≈ 10–15%), illite (≈ 3–5%), and minor calcite and quartz impurities. The Bełchatów clays exhibit liquid limits (*Wl*) in the range of 92–122%, plastic limits (*Wp*) of 26–38%, and plasticity indices (*Ip = Wl – Wp*) between 61 and 87%. Such values classify them as highly plastic, highly active clays (activity index *A* ≈ 3.4), consistent with their beidellitic mineralogy and fine particle size^132–134^.

A cation exchange capacity (CEC) between 317 and 574mval/kg (≈ 32–57cmol(+)/kg), with exchangeable cations dominated by Ca²⁺ > Mg²⁺ > K⁺ > Na⁺. This exceptionally high CEC reflects the abundant negatively charged layer sites typical of smectitic structures and correlates with high surface hydration and interparticle repulsion. These characteristics are directly relevant to the rheological behavior of the clay–cement suspensions studied here: higher CEC and plasticity indices correspond to greater water adsorption, higher yield stress, and enhanced thixotropy. The strong electrostatic interactions and interlayer water structuring of beidellite promote the formation of a cohesive, reversible particle network, which governs both the viscoelastic response (G′/G″ ratio) and flow properties of the fresh suspensions^133,135^.

### Activator

A sodium silicate solution (designation R145) is and aqueous solutions of sodium silicates with molar modulus SiO_2_/Na_2_O as 2.4/2.6 and oxide content (SiO_2_ + Na_2_O) [%], not less than 39%.

### Mixture design and preparation

Suspensions were prepared by first creating a homogeneous clay-water mixture at the specified density, followed by controlled addition of the solid components (in order: cement, fly ash, and sodium silicate when applicable) under continuous mechanical mixing to ensure uniform dispersion and prevent agglomeration. Mixing was conducted using a two-head hand mixer operating at 500 rpm, which provided sufficient shear to integrate the solids without inducing excessive air entrainment.

## Methods

### Chemical analysis and mineralogy

The elemental composition of the samples was determined using a wavelength-dispersive X-ray fluorescence spectrometer (Bruker S8 TIGER). Measurements were carried out under vacuum conditions employing the instrument’s Quant Express standard calibration module. The results were used to identify and quantify the principal oxides present in the investigated materials.

The crystalline phases were identified by X-ray diffraction using a PANalytical Empyrean diffractometer equipped with a Cu Kα₁ radiation source (λ = 1.54178 Å). Diffractograms were recorded over the 2θ range of 5–90°, with a step size of 0.008°. Phase identification was performed using the PANalytical HighScore Plus software package in conjunction with the PDF-2 (2004) and ICSD (FIZ Karlsruhe, 2012) reference databases.

### Textural properties and density

The specific surface area and skeletal density were determined using a Micromeritics ASAP 2010 analyzer. Prior to analysis, the samples were degassed under vacuum (10⁻³mmHg) at 100 °C for 24 h to remove adsorbed moisture and gases. Nitrogen adsorption–desorption isotherms were obtained at liquid nitrogen temperature using N₂ of 99.999% purity as the adsorbate. The BET multipoint method was applied to calculate the specific surface area within the relative pressure range of 0.05–0.30. Instrumental volume calibration was conducted immediately prior to measurement to ensure accuracy.

### Rheological characterization

#### Rotational rheometry

**Fig. 4:**
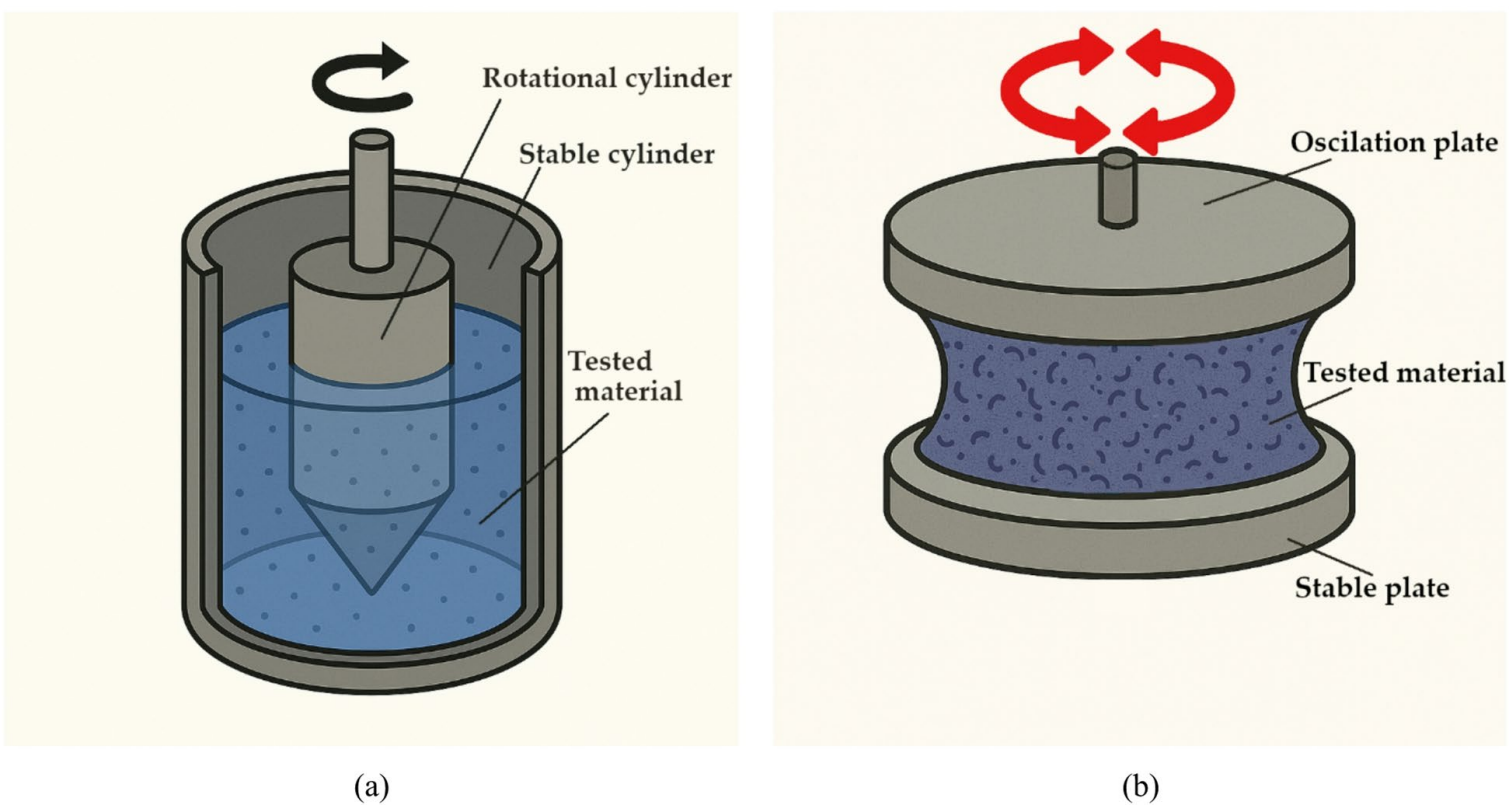
Rheology analysis methods (**a**) coaxial cylinders shear measurement; (**b**) plate to plate oscillation measurement. *Image generated with DALL·E 3 (OpenAI*,* March 2025 build) from an original prompt; included for illustrative purposes only*,* no data derived from this image. Licensed CC BY 4.0.*

Flow behavior and viscosity profiles were assessed using a Brookfield DV-III + rheometer equipped with a TC 500 thermostatic system. Measurements were conducted using coaxial cylindrical geometry at controlled temperature (22 ± 1 °C). Schematic presentation of measurement system is presented in Fig. 4a.

**Fig. 5:**
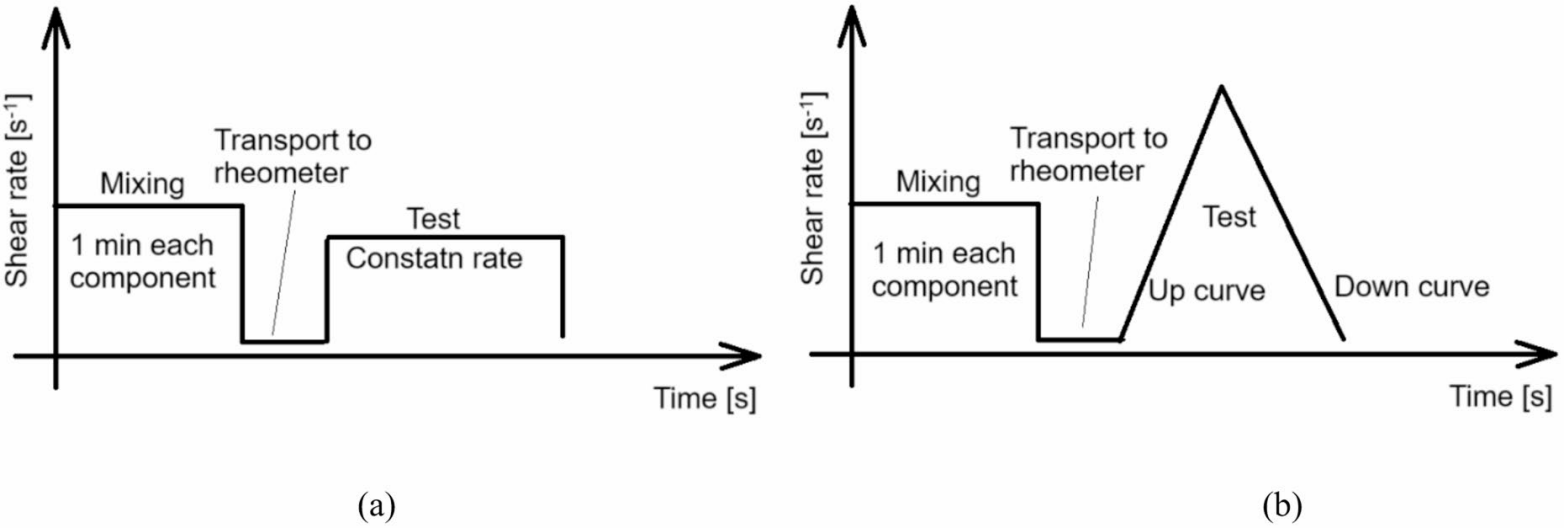
Schematic representation of rheological test procedures: (**a**) constant shear rate; (**b**) flow curve determination through increasing and decreasing shear rate ramps.

Reactivity profiles where obtained in constant shear rate 20^−1^s measurements (Fig. 5a). Flow curves where obtained by increasing rump phase - gradually increasing shear rate from 5 to 50s⁻¹ (20 min), followed by descending ramp phase - gradually decreasing shear rate from 50 to 5s⁻¹ (20 min), with total time of measurement 40 min (Fig. 5b).

Those procedures enabled quantification of:

- Reactivity profiles.
- Flow curves (shear stress vs. shear rate).
- Apparent viscosity profiles.
- Thixotropic behavior through hysteresis loop area calculations.
- Equilibrium yield stress using the Bingham model applied to the stable flow region.
- (~ 20–40 s⁻¹)
- Power of structure destruction/rebuilding, calculated as an integration of the area between flow curves for 1-second segments^12,136–138^.

#### Oscillatory rheometry

Viscoelastic properties were characterized using an Anton Paar Physica MCR 301 rheometer with a plate-to-plate measurement system (25 mm diameter plates, 0.3 mm gap). Schematic presentation of measurement system is presented in Fig. 4b.

Frequency sweep tests were conducted to evaluate the dynamic mechanical response with constant strain amplitude of 3% (within the linear viscoelastic region), frequency range: 0 to 150 Hz with rate of 1 Hz rise each 16 s (40 min to reach 150 Hz), temperature maintained at 22 ± 1 °C.

From oscilation measurements, the following parameters were determined:

- Storage modulus (G′): representing the elastic component.
- Loss modulus (G″): representing the viscous component.
- Viscoelastic ratio (G′/G″): indicating the dominant rheological behavior^12,139,140^.

#### pH measurement procedure

pH measurements were performed during selected tests to monitor the early chemical evolution of the mixtures and to relate pH variations to the observed rheological behaviour. Immediately after mixing, a calibrated pH electrode was immersed directly into the fresh suspension, ensuring continuous contact between the sensing tip and the homogenized sample. Measurements were recorded at defined time intervals throughout the first hour of the experiment, capturing the pH changes associated with dissolution, ion release, and early-stage reaction processes.

#### SEM and microstructural analysis

Microstructural observations were carried out using Scanning Electron Microscopy (SEM) on after 30 and 60 min after mixing. Hydration was stopped through a solvent exchange method, by adding 2-propanol (isopropyl alcohol) into mixtures at resp. time.

Imaging was performed with a Thermo Scientific Phenom XL microscope operated under high-vacuum conditions. The analysis focused on identifying morphological features, assessing particle geometry, and evaluating the development of the hydration and consolidation processes within the hardened matrices.

#### Isothermal calorimetry

Heat release was measured using a TAM Air isothermal microcalorimeter at 25 °C for approximately 60 h (only 28 h time window is presented in order to improve readability). Pastes were prepared by mixing 3 g of binder (cement with the specified fly ash and clay fractions 50:50%) with 1.5 g of distilled water. The components were homogenized for 30 s in a vibration mixer, after which the fresh paste was transferred into a 20mL sealed glass ampoule. The interval between water addition and measurement start was maintained at 90s. A quartz-sand reference ampoule was used to match heat capacity. The calorimeter provides high-stability measurements with a detection limit of 2µW and temperature accuracy better than ± 0.15 K.

#### Mixture formulations

The study employed a systematic approach to mixture formulation to explore complete rheology of clay-cement sealing suspensions with fractionated fly ashes.

Firstly, the reactivity of each fraction with water was studied. Constant shearing with rate of 20s^− 1^ was applied to water/ash (w/a) suspensions with ratio: S1-10/3 and S2-1/1. Next, four main parameter variation studies were conducted:

- Base suspension density effect (1.13–1.22 g/cm³) with fixed composition of 20% fly ash, 10% cement, sodium silicate (R145) 0% by wt;
- Fly ash content variation (5–40% by wt), base density 1.20 g/cm^3^, R145 0%; and cement 10% by wt;
- Cement-fly ash substitution with ratios of 10/0, 8/2, 5/5, 2/8, and 0/10 at constant 10% total solid content and R145 0.5%, density 1.20 g/cm^3^;
- R145 content (0%, 0.5%, 1.0%, 1.5% by wt), density 1.20 g/cm^3^, cement/ash ratio 5/5 at constant 10% total solid content, with all measurements conducted at 22 ± 1 °C.

Rheological data were analysed using manufacturer software and further processed with custom algorithms to derive rheological parameters.

## Results & discussion

### General rheological properties

#### Fly Ash reactivity and initial viscosity development

**Fig. 6:**
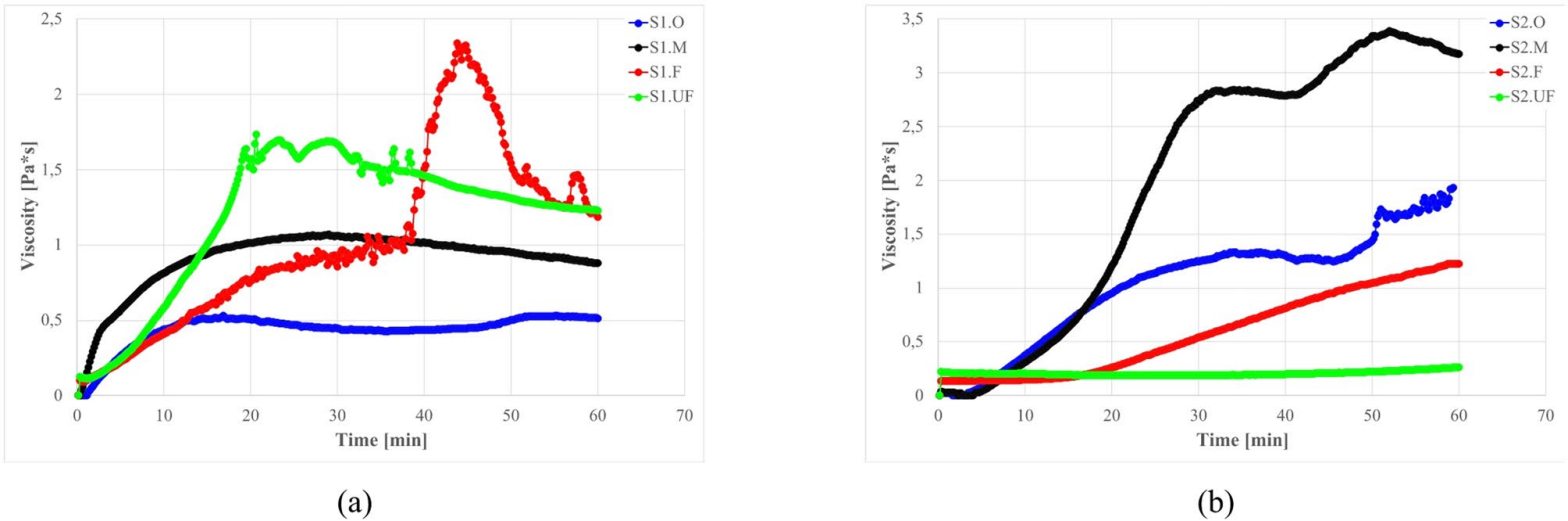
The influence of ash fraction on the viscosity of water-ash suspensions; fly ash short-time reactivity, shear rate 20s^− 1^, water/ash (w/a) ratio: (**a**) S1- 80/20%; (**b**) S2–50/50.

The rheological behavior of suspensions containing fly ash particles is significantly influenced by the chemical composition, particle morphology, and size distribution of the ash fractions. To isolate the short-term reactivity of each ash fraction, fly ash–water suspensions (no clay or cement) were examined under a constant shear (20 s^− 1^). Figure 6 compares the evolution of apparent viscosity for S1 (high-Ca) and S2 (high-Si) ashes and their fractions in water suspensions under constant shear rate 20s^− 1^.

The S1 ashes, and especially ultra fine (S1.UF) fraction, showed rapid viscosity increases after mixing within 20–50 min. The high calcium content in S1 ash (25.4–37.8% CaO for S1.O and S1.UF resp.) and reactive mineralogy (high anhydrite content, Ca-Al and Ca-S-Al oxidesa) provides similar ionic driving forces similar to sufflaminate cements, where calcium aluminate/sulfate phases dissolve within minutes, creating ion supersaturation that leads to ettringite and C–S–H precipitation shortly after mixing (~ 5 min)^141–143^. The abundance of Ca²⁺ and SO₄²⁻ ions alters the suspension’s electrochemical environment, creating immediate electrostatic particle bridging, promoting aggregation and causing structure build-up^56,143–146^. The immediate availability of sulfate (from S1 anhydrite) and alumina (from glassy phase) leads to early ettringite crystallization, which contributes to stiffening by forming a needle-like crystalline network in the mix. This induces coagulation of fine-sized ash particles into a space-spanning network within minutes.

After the initial activation period, the system stabilizes within approximately 30–40 min. The emergence of a secondary viscosity peak can be attributed to the attainment of a critical ionic concentration, which promotes the formation of ettringite^93,147,148^ and early hydrate gel formation^141,143^, particularly the nucleation of calcium silicate hydrate (C-S-H) and calcium aluminate hydrates (C-A-H). These processes indicate the pronounced self-cementing and pozzolanic activity of the high-calcium S1 ash^45,88,117^.

Particular grains seems to be too heavy to remain suspended in water and undergo relatively fast sedimentation, while for S1.O this sedimentation is prolounged by appearance of fine grains, which due to its low settling velocity and high reactivity allows to formation of a special gell-like high viscous structure with which “freeze” mixture in suspended form. Coarse fractions in such system may remain suspended or undergo slower sedimentation, which in combination of reactive component result in stabilisation of S1.O viscosity curve after around 10 min with only small viscosity sweep at around min 50^88,129^.

**Fig. 7:**
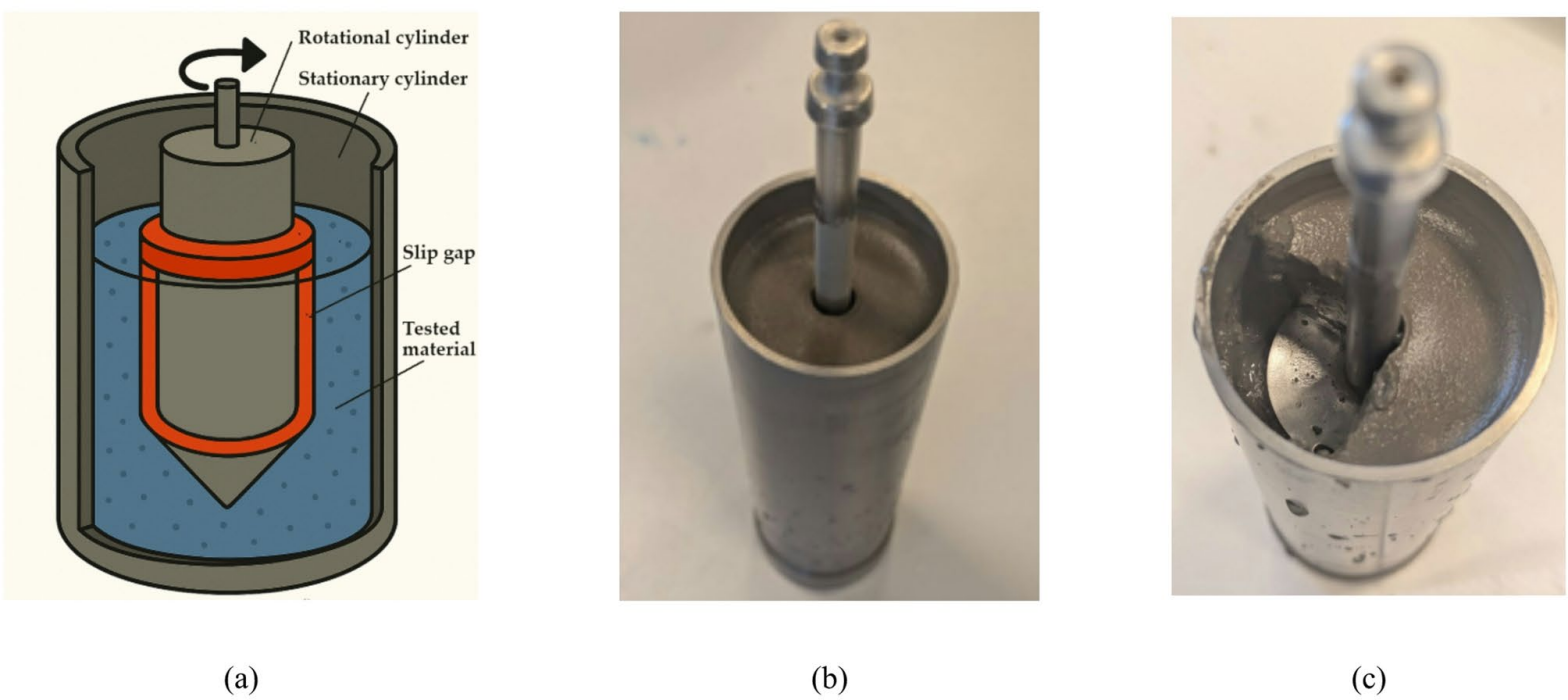
Disturbances during rheological measurements (**a**) slip gap scheme in coaxical cylinders system; (**b**) post measurement photo of S1.UF ash-water 2–8 ratio mixture; (**c**) crossection with slip gap. *Image a) generated with DALL·E 3 (OpenAI*,* March 2025 build) from an original prompt; included for illustrative purposes only*,* no data derived from this image. Licensed CC BY 4.0.*

 For the S1.UF fraction, a paradoxical combination of high chemical reactivity and measured viscosity drop after 45 min was observed. This behavior is primarily attributed to the ultrafine particle size, which produces an exceptionally smooth shear interface and promotes the formation of a slip gap between the sample and the rheometer surface. The ultrafine ash fraction contains a high proportion of amorphous, glassy material enriched in network-modifying cations (Ca, Mg, Na, K), which rapidly dissolve after mixing, initiating hydration and early gel precipitation. As previously described in literature^149–151^, during the early-age hydration of cementitious systems the precipitated C–S–H and other gel/solid phases nucleate preferentially at particle surfaces and contact zones. Thus, the bulk of the material (i.e., the inter-particle suspension region) can undergo bulk stiffening even while the rheometer records the shearing of a thin boundary layer, especially for finer fractions. Under these conditions, the rheometer records only the shearing of a thin, lubricated boundary layer—rather than the bulk flow of the suspension—leading to an apparent reduction in viscosity. Thus, the observed “low viscosity” reflects a measurement artifact associated with slip-gap formation rather than genuine fluid mobility. This artifact arises from the combination of high fineness, smooth particle morphology, and fast-reacting glassy phases that collectively produce a super-slippery interface and isolate the bulk from the applied shear field. A representation of this effect is provided in Fig. 7^152–155^..

The formation of a slip plane in S1-high systems indicates extreme flocculation: a solid-like mass forms in the bulk, which no longer shears homogeneously (the structure is too rigid), indicating transition from viscoelastic to partially solidified system with heterogeneous properties. This signals application challenges and opportunities—rapid structuration with premature setting and loss of pumpability, while can be useful when fast structuration is desirable. Proper proportions must be controlled to prevent overdosage and undesired stiffening^156–158^ and with S1 finer fractions initiate rapid structuration, while coarser fractions contribute through physical particle interactions^24,40,77,88,129,159,160^.

In contrast, S2 (siliceous) ash suspensions remain fluid and workable for an extended period, displaying only a gradual viscosity increase over the testing period. Initially, S2 acts as an inert filler – its smooth, glassy microspheres (predominantly SiO₂–Al₂O₃) reduce inter-particle friction^49,69,89,161^. The absence of abundant Ca-bearing phases precludes early cementitious reactions (calcium content of S2 ashes CaO: 4.2–6.6% vs. 25.4–37.8% for S1 ashes). Directly after mixing within minutes, a slow upward drift in viscosity occur, which is attributed to sedimentation. In case of S2.M fraction stabilisation occurs around 30 min suggesting on fast densification of bottom layers and formation of slip gap, while for finer fractions with lower sedimentation velocity this process is more prolonged in time.

Additionally, weak alkali–silica dissolution may also influence respond behavior. The S2 ashes contain slightly elevated K₂O and MgO (e.g. K₂O ~ 3%, MgO ~ 2.5–4%, Table 4) that can be leached out in alkaline water. Such dissolution, in combination with cement hydration products may create a conditions, casing slow structuration/flocculation, These species gradually break down the silicate glass network, releasing silicate ions that form an alkali-silica gel, hovewer, this effect might be observed only in longer timescale of hours or days^46,162–164^. The increase of pH is promoting this effect even in ambient conditions, and will be discussed later. The resulting gel induces a gentle structuration of the suspension (a slow viscosity rise), though much less pronounced than the cementitious-like setting seen in S1.

It is worth noting that S2 fine fractions also contain a high fraction of unburned carbon (LOI 7–13% vs. 1–2% for S1 ashes), which remains chemically inert in water but can slightly alter rheology by adsorbing admixtures or water. High LOI coal-fly-ash residuals are often considered inert in terms of hydraulic reactivity; however, the elevated unburned-carbon content can significantly influence the fresh rheology of cementitious systems by adsorption of admixtures and water, and by altering particle packing and surface chemistry^165–167^. For instance, Wu et al.^168^ observed that high-LOI fly ash additions increased yield stress and plastic viscosity, owing to elevated water demand and poorer superplasticizer efficiency.

**Fig. 8:**
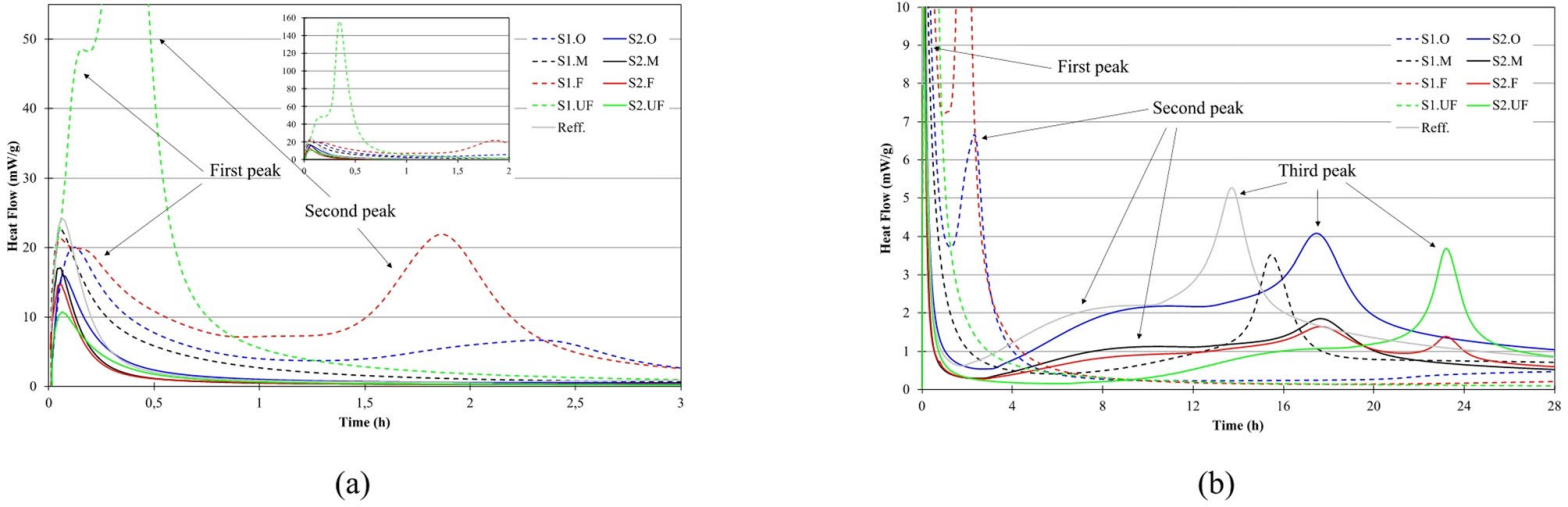
The influence of ash fraction on normalized heat flow of water-cement-ash mixtures; (**a**) 3 h time window (small picture on top as 2 h window); (**b**) 28 h time window; water/binder (w/b) ratio 0.5; cement/ash ratio 50/50%; reff. sample - cement 100%.

#### Fly Ash reactivity in Water-Cement mixture. Isotermal calorimetry

The isothermal calorimetry profiles (Fig. 8) shows markedly different early hydration behavior between S1 and S2 ashes, strongly correlated with their chemo-mineralogical composition. In the case of S2 (siliceous) ashes, the initial heat flow is notably lower than that of the cement reference, with values decreasing with increasing fineness (~ 17mW/g for S2.M and ~ 10mW/g for S2.UF vs. ~24mW/g for OPC). Despite higher surface area and amorphous content in finer S2 fractions compared to coarser, their early reactivity remains low, likely due to the predominance of inert silica, high LOI, and the delayed solubility of glassy phases at this early stage. This behavior confirms that S2 primarily acts as a dilution agent rather than a reactive component during the first hours. The heat profile of these mixtures shows only a brief initial signal (likely from early C₃A–gypsum interaction in the cement), followed by secondary phase. The delay in secondary heat release (~ 4–12 h) is longer for finer S2 fractions, suggesting restricted ion transport or retarded activation—possibly tied to low pH rise and slower ion availability. This correlates with improved early workability and delayed structuring in S2-based mixtures, as confirmed in rheological observations.

In contrast, S1 (Ca-rich) ashes demonstrate pronounced early activity, with initial heat flow values (~ 22–23mW/g for S1.F/M and ~ 48mW/g for S1.UF) comparable to or exceeding OPC. The high surface area and abundance of reactive Ca-, Al-, and SO₃-bearing phases in S1 enable rapid dissolution and early precipitation of ettringite and potentially C–S–H or C–A–H gels. Particularly in S1.UF, a sharp second exothermic peak (~ 160mW/g) emerges within 0.5 h, indicative of intense early-stage reactions. This behavior parallels the hydration kinetics of calcium sulfoaluminate cement (CSA) with rapid setting within minutes after contact with water^141–143^. In CSA-type cements, the early sulfate–aluminate reactions drive quick ettringite formation and early structure buildup^169,170^. The continuation of elevated heat flow beyond the initial peak—especially in coarser S1 fractions—suggests sustained hydration, without a distinct dormant phase. This sustained release implies continuous solid phase formation and is consistent with rheological findings of rapid, multi-stage stiffening in S1-rich systems.

Interestingly, for the finest S1 fractions (especially S1.UF), the expected third hydration peak—typically linked to diffusion-controlled silicate hydration^171^ —is less pronounced or absent. This can be attributed to early formation of a dense microstructure that restricts ionic mobility and pore solution exchange. Rapid gelation and precipitation around reactive particles likely immobilize water and limit further dissolution or precipitation processes. Such behavior is analogous to CSA pastes enriched with silica fume or ultrafine additives, where rapid microstructure closure suppresses later-stage heat evolution as was also mentioned by Zhang et al.l. while exploring fly ash, silica fume and blast furnace slag influence on hydration kinetics of OPC and CSA^171,172^. In this way, early reactivity paradoxically reduces long-term exothermic activity, steering the system into a premature retardation phase. Overall, the calorimetric patterns reinforce the rheological picture: Ca-rich S1 systems form solid networks early via rapid hydration, while siliceous S2 systems preserve fluidity through minimal early reactions—offering complementary pathways depending on performance targets.

#### Clay-cement-ash suspensions

##### Initial viscosity development of clay-cement suspensions with ash additives

**Fig. 9:**
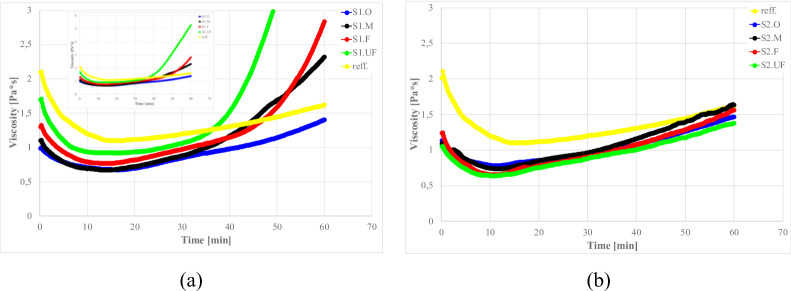
The influence of ash fraction on the viscosity of clay-cement-ash suspensions; short-time reactivity, shear rate 20s^- [1^, FA 5% Cem 5% 1.20 g/cm^3^, R0.5%, (**a**) S1 ashes, (**b**) S2 ashes.

For clay–cement suspensions containing 5% cement and 5% fly ash at a total solid load of 10% (Fig. 9, the early response is dominated by the clay network, which undergoes shear-induced breakdown during the first ~ 10 min and viscosity drops to 0,93 Pa·s in S1.O and 0,68 Pa·s S2.O systems resp. The high-Ca S1 ash rapidly releases Ca²⁺ which exchange with native cations in the clay (e.g., Na⁺ in bentonite). According to the theory, this cation exchange compresses the clay’s diffuse double layer, causing the clay platelets to flocculate. In essence, Ca²⁺ acts as a bridge between negatively charged clay surfaces, rapidly converting the clay from a dispersed to an aggregated structure. In contrast, the siliceous S2 ash introduces mainly monovalent ions (K⁺, Na⁺) and very little Ca²⁺. Monovalent cations do not effectively flocculate clay – on the contrary, a Na⁺- or K⁺-rich environment tends to keep clay particles dispersed^173,174^. Thus, S2 suspensions retain a more open, fluid structure due to the lack of divalent bridging cations and tends to disperse the clay matrix rather than build structure. Additionally, smoother and more spherical S2 ashes reduce interparticle friction (ball-bearing effect), leading to lower yield stress and viscosity.

**Fig. 10:**
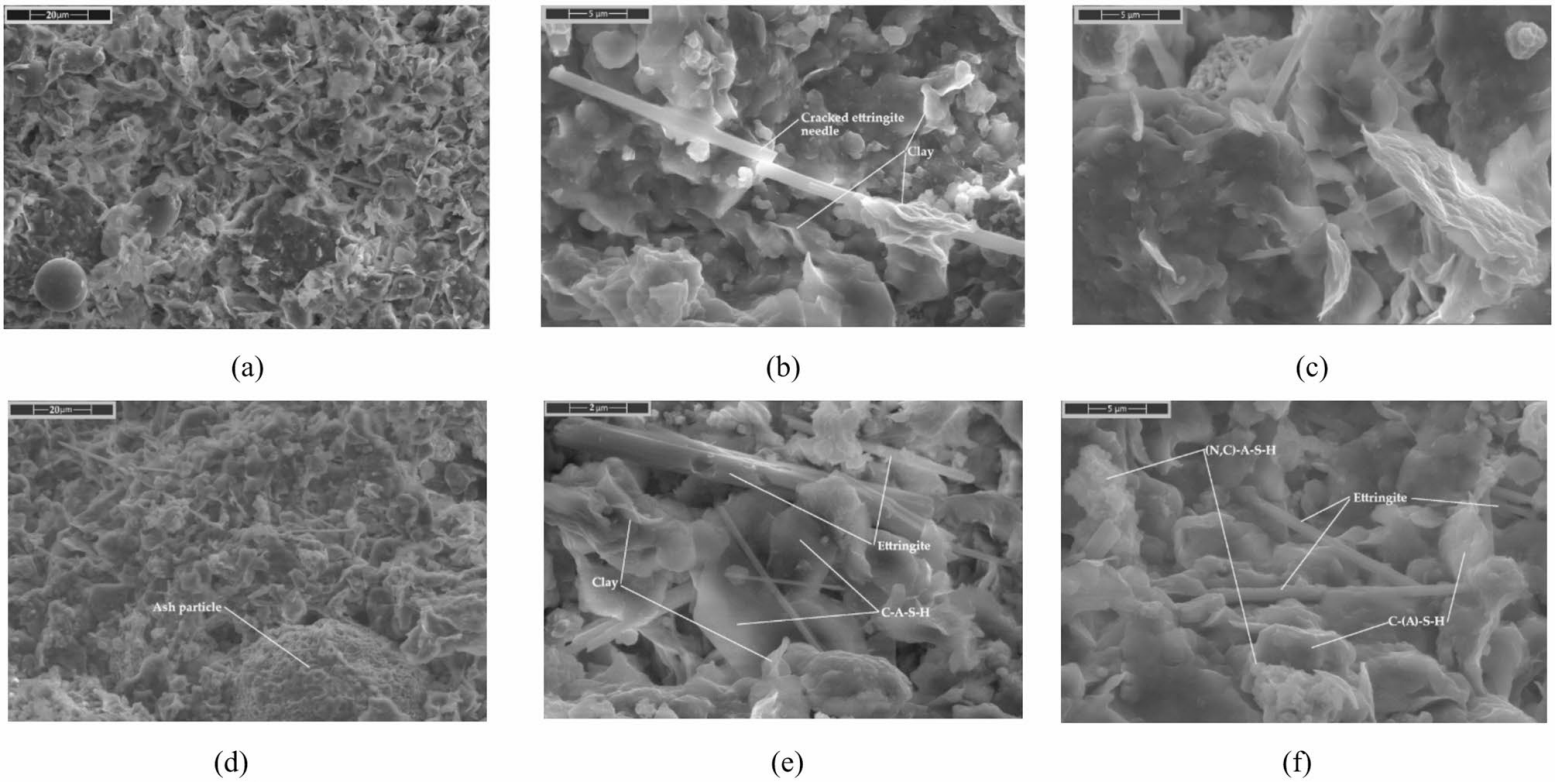
SEM images of microstructure at (**a-c**) 30 min and (d-f) 60 min after mixing for S1 ash mixtures. FA 5% Cem 5% 1.20 g/cm^3^, R0.5%.

**Fig. 11:**
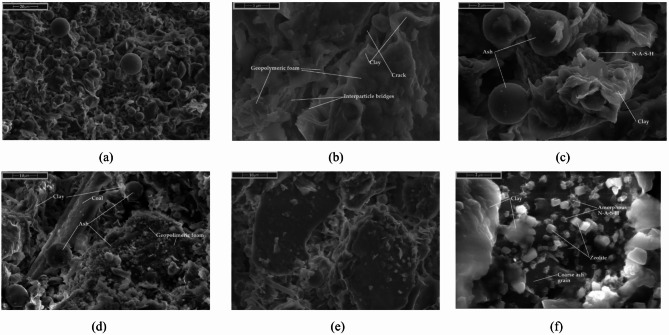
SEM images of microstructure at (**a-c**) 30 min and (**d-f**) 60 min after mixing for S2 ash mixtures. FA 5% Cem 5% 1.20 g/cm3, R0.5%.

The formation of various hydration products—such as N-A-S-H, C-A-S-H, C-S-H, and C-N-A-S-H, both amorphous and crystalline—can occur simultaneously under suitable conditions. Evidence of these early phases, along with clay particle structuration, is visible in the presented SEM images at Figs. 10 and 11^173–177^.

SEM images shows how microstructure evolves within the first hour depending on ash type. In S1 mixtures, the high Ca²⁺ content promotes early flocculation and rapid precipitation of ettringite and C–S–H, forming dense, interlocked structures even at 30 min. By 60 min, the network thickens visibly with needle-like hydrates bridging clay and ash particles, increasing stiffness.

In contrast, S2 systems remain more open and dispersed due to low calcium and monovalent ion-dominated chemistry. At 30 min, smooth ash spheres are mostly free; by 60 min, a thin amorphous gel layer (likely N–A–S–H or C–N–A–S–H) starts to appear, along with possible zeolitic phases. The structure is still weak and gelation remains incomplete, correlating with better flow retention.

**Fig. 12:**
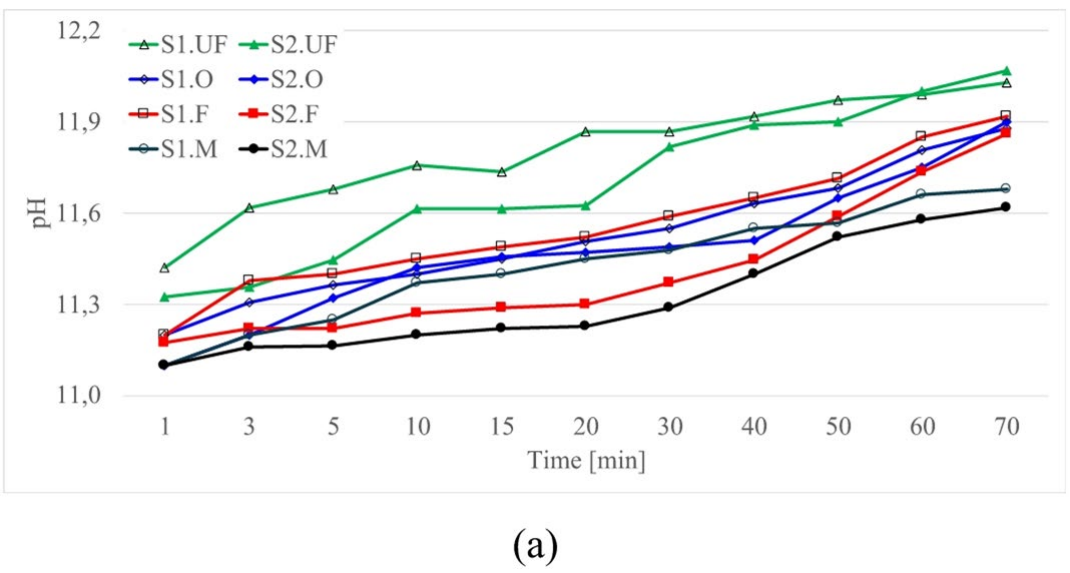
The time dependent change of pH in clay-cement-ash suspensions. S1 and S2 ash mixtures: FA 5% Cem 5% 1.20 g/cm^3^, R0.5%.

Complementary pH monitoring confirmed a progressive increase in alkalinity corresponding to the timing of viscosity transitions (Fig. 12). In S1-based systems, pH increased from approximately 11.3 to 11.9 within the first 30 min and stabilized near 12.0 after 50 min, reflecting continuous dissolution of reactive phases and precipitation of early hydrates. In contrast, S2 systems exhibited an initial pH of ≈ 11.2 that rose gradually to 11.6 after 30 min and further to 12.0 after one hour. Finer fractions have higher initial pH values as well as more pronounced pH increase in time. Surpassing the pH threshold of ≈ 11.5 appears to trigger secondary reactions associated with the dissolution of aluminosilicate phases and creating conditions to geopolymerisation under ambient conditions^114,177–180^.

The S2-based mixtures (Fig. 9b) display a uniform trend across fractions: an initial shear-induced thinning phase, followed by a slow and steady viscosity rise. In these systems, the rate of increase is greatest for the coarse (M) fraction, likely due to more rapid sedimentation of heavier particles and the associated densification of the suspension. Finer S2 fractions maintain lower viscosity for longer, benefiting from their greater packing efficiency and reduced particle interlocking, which preserve fluidity. The gradual thickening is attributed to cement content and its time dependant hydration.

Cement in those mixtures dissolves and hydrates shortly upon mixing, releasing Ca(OH)₂ that raises the pore water pH. This alkaline surge accelerates the dissolution of fly ash glassy phase and provides additional calcium and sulphate ions inducing thickening. Ylmén et. all^150^ described this “flesh setting” as background ionic strength increase and intermediate ettringite, monosuplhate and polymerized silica phases formation. In S1 ash mixtures, the abundance of calcium and sulphate sources (both cement and ash, especially in fine fractions), promote such “flesh setting” behavior.

The rheological response of cement–ash suspensions arises from the evolving microstructure controlled by interparticle bonding and water distribution. As described by Li et al. (2024), stress transmission occurs through transient C–S–H bridges that repeatedly rupture and reform between neighbouring grains, creating a dynamic network of “virtual bonds.” The frequency and stability of these contacts—governed by particle size, packing density, and hydration kinetics—determine the yield stress and thixotropic recovery. Concurrently, the relative proportions of bound, flocculated, and free water dictate particle mobility: bound water integrates into hydrates, flocculated water resides in cluster voids with minimal rheological effect, while free water lubricates the shear interface. Reactive or fine ashes reduce free water through adsorption and early gel precipitation, thereby densifying the microstructure and increasing resistance to flow. Hence, the apparent rheology reflects the competition between bond rupture–reformation dynamics and the progressive immobilization of free water within the hydrating particle network. Finer particles or denser packing enhance the frequency of such contacts, promoting thixotropy and structural recovery, whereas coarser or poorly packed systems exhibit weaker stress transmission and lower cohesion.

#### Base suspension density effect on rheological properties

**Fig. 13:**
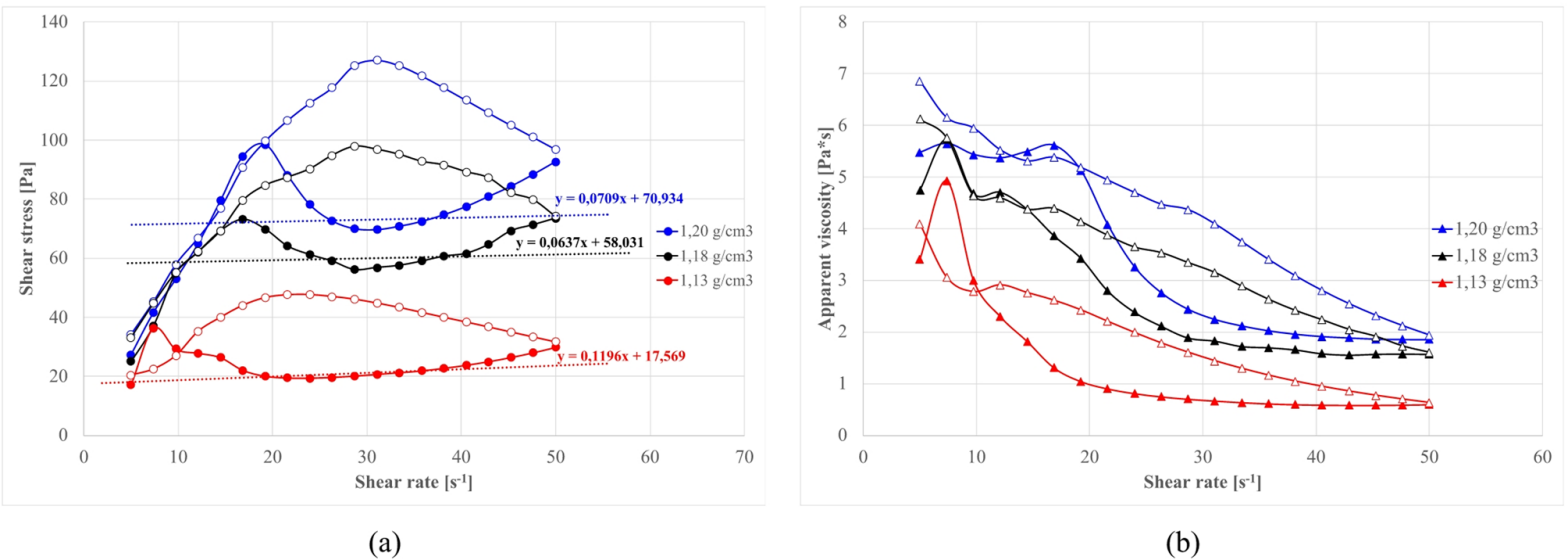
Effect of base suspension density on the properties of waterproofing cement slurries as a function of shear rate: (**a**) flow curves, (**b**) apparent viscosity. Formula: S1.O 20% by wt., cem. 10%, R145 0%.

**Fig. 14:**
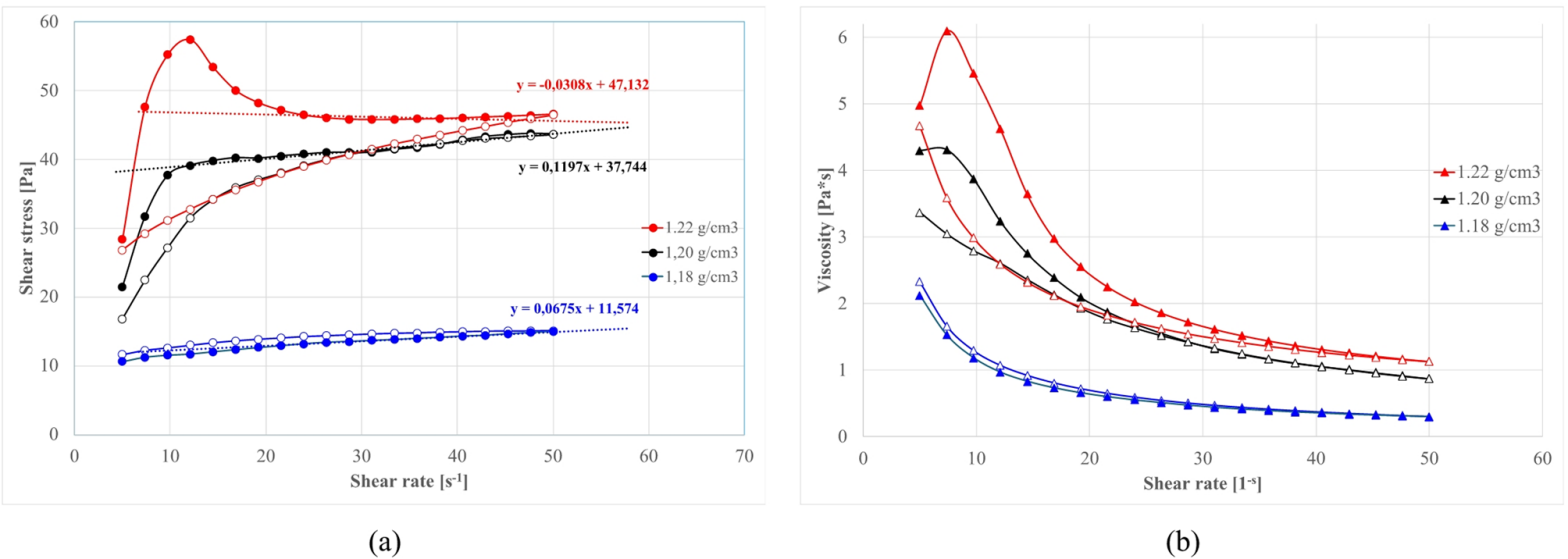
Effect of base suspension density on flow curves. Effect of base suspension density on changes in apparent viscosity as a function of shear rate. Formula: S2.O 20%, cem 10%, R0%. (% by wt.).

Having established baseline ash behaviors, the study next examined full clay–cement–ash suspensions. Figures 13 and 14 shows how the base suspension density (solids concentration of the clay–water mix before cement and ash addition) affects rheology for formulations with S1.O and S2.O ashes, respectively.

As expected, raising the base density from 1.13 to 1.22 g/cm³ (i.e. using more clay per unit water) increases viscosity and yield stress across all mixtures. Higher density slurries contain less free water and more clay particle contacts, resulting in steeper flow curves (greater shear resistance) due to intensified inter-particle friction and flocculation^22,26,181–183^.

The observed flow curves was related to the Bingham model which is commonly used for the characterization of cement pastes. Based on this model, the slope of the linear segment of the flow curve represents the plastic viscosity, while its intersection with the stress axis indicates the Bingham yield stress^12,49,184^. For example, in S1.O systems the Bingham yield stress climbs from ~ 19 Pa at 1.13 g/cm³ to ~ 71 Pa at 1.22 g/cm³ (with 10% cement, 20% ash). S2.O mixtures shows a similar trend (e.g. ~12 Pa to ~ 42 Pa over the same density increase). This quantifies the strong influence of the base suspension influence: a denser clay matrix markedly stiffens the fresh slurry, combining both effects of the colloidal clay network and reduced lubrication water thickening effects, which effectively means a higher degree of particle crowding and potential flocculation.

Worth noticing, these multi-phase systems with clay, cement, and fly ash interactions exhibit non-linear rheological behavior that the used Bingham model cannot fully capture, particularly at low shear rates critical for these applications, so can only be used as partial analytical tool.

The influence of fly ash type remains consistent across densities: S1 fractions consistently accelerate structural build-up compared to S2 fractions. While higher densities elevate baseline rheological parameters, the relative effects of ash characteristics on workability remains independent of suspension density^185,186^.

Flow curves shows characteristic clay-based behavior: initial stress growth at low shear rates followed by sudden decrease at 5–20 s⁻¹, indicating three-dimensional initial clay network breakdown pseudoplastic behavior^12,181,182^. Higher base density shifts this inflection toward higher shear rates and increases overall stress, suggesting more robust networks requiring higher shear rates and greater disruption forces to be disrupted^187–189^.

S1.O ashes shows negative thixotropic behavior with descending curves exceeding ascending ones due to high ash reactivity and enhanced time-dependent structural iridization^21,138^. This is related to the clay and ash particles flocculation into a particle network due to Ca²⁺ bridges and to early hydrate precipitation, which add to gradual shear stress increase over time. Such flocculation manifests as an increase in yield stress over time (structural build-up)^129,190^.

After initial structure breakdown (approx. 20s^- 1^), in S1 ash systems the secondary stress rise (starting in approx. at 30s⁻¹, Fig. 13a) indicates early hydration onset, as was discussed in earlier. S2.O ashes exhibit shear thinning behavior with characteristic inflection near 10s⁻¹ marking structural breakdown^12,189^. Compared to rapidly evolving S1 systems, S2 suspensions maintain stable flow behavior suitable for applications requiring rheological consistency. The limited amount of strong flocculating results in a dispersed system forming, where clay platelets remain largely separated. Dissolved species from S2 (e.g. monovalent Na⁺, K⁺) do not induce particle bonding, which preserves the dispersed state. Instead of forming a gel structure, S2 ashes in suspensions behave as stable dispersions, showing lower yield stress.

#### Effect of fly Ash content on rheological properties

**Fig. 15:**
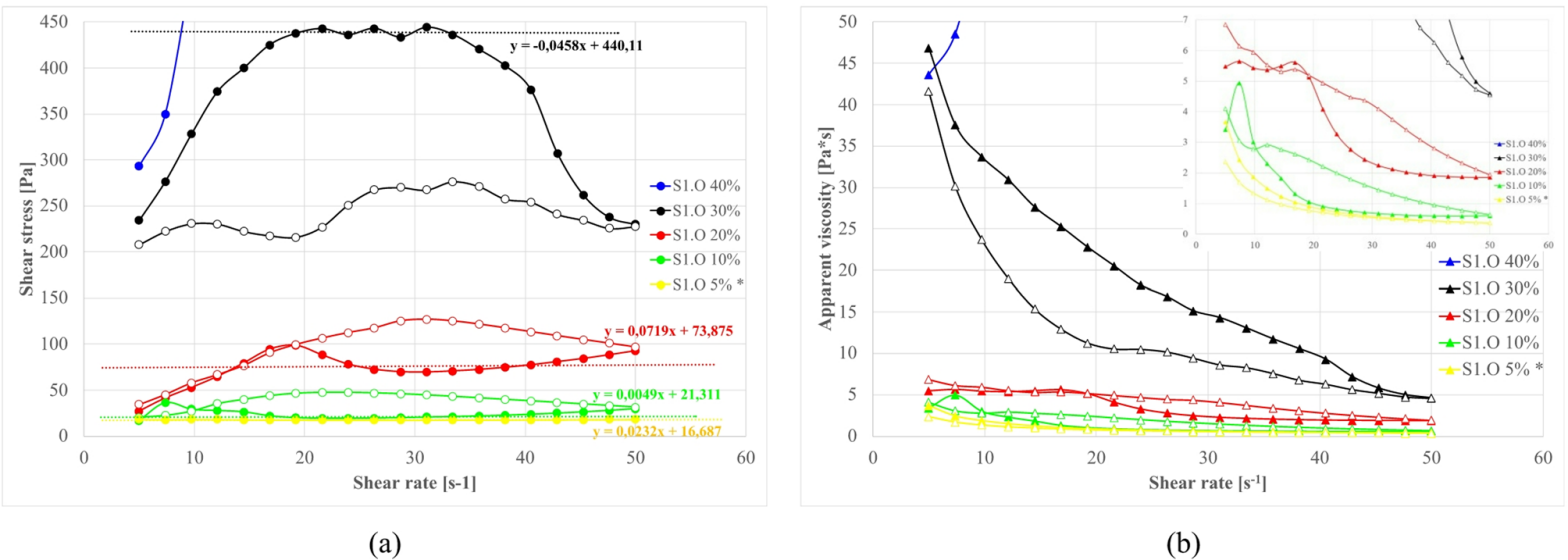
Effect of S1.O ash total load (% wt) on properties of sealing suspensions as a function of shear rate: (**a**) flow curves, (**b**) apparent viscosity. Formulas: base density 1.13 g/cm³, cem. 10%., R145 0%. *base density 1.13 g/cm³, cem. 5%, FA 5%, R145 0%.

**Fig. 16:**
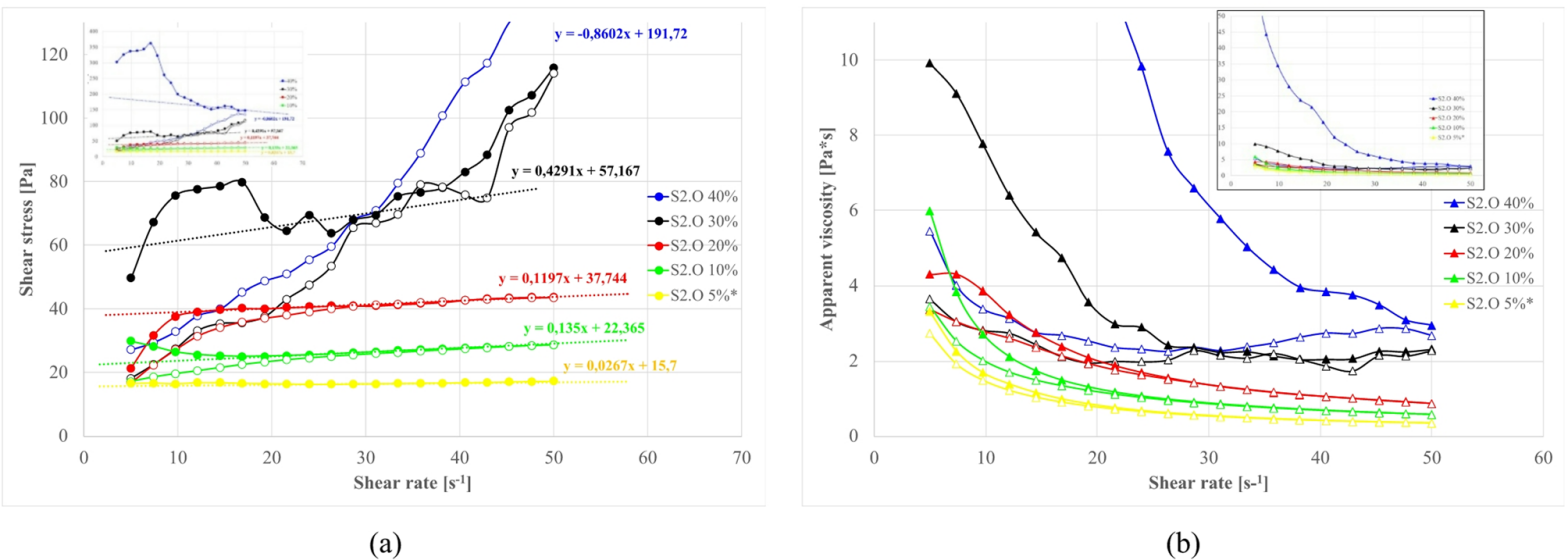
Effect of S2.O ash total load (% wt) on properties of sealing suspensions as a function of shear rate: (**a**) flow curves, (**b**) apparent viscosity. Formula: S2.O 1.20 g/cm³, cem 10%, R0% *base density 1.20 g/cm³, cem. 5%, FA 5%, R145 0%.

The influence of total binder content – particularly the dosage of fly ash added – is investigated in this chapter. Figure 15 (S1 series) and Fig. 16 (S2 series) present flow curves and viscosities for suspensions with increasing ash fraction (while cement is fixed at 10%, clay fraction adjusted to maintain base density). In order to avoid premature stiffening in mixtures with higher dosages of S1 ashes, and to allow wider range of testing, the 1.13 g/cm^3^ base density was used. For S2 ashes however, the 1.20 g/cm^3^ base density was applied.

At low S1 ash content (5–20% of total mass), the flow curves maintain a thixotropic loop with descending curve below ascending. This phenomenon is distinct from pure clay-induced thixotropy (where structure firstly breaks down under shear and rebuilds at rest during descending cycle), in this clay-cement-S1.ash systems the structure builds up assigned to time related chemically induced thickening that outpace shear-induced mechanical breakdown. Unlike classical rheopexy (shear-thickening due to orientation/structure under shear observed in gypsum or some bentonite suspensions), the observed shear stress increase is caused by ongoing hydration/flocculation in the suspension with internal structure development and successive shear stress increase^191–193^. The effect depends on reaction time rather than shear history.

S1.O systems at higher ash concentrations 30–40% are shaped mostly by ash contribution. Excessive ash dosage causes excessive water absorption, extreme gelation with fluidity loss and brittle solid-like behavior of mixture, confirming critical solid loading thresholds resulting in pronounced impact on rheological response^12,27,189^. The inverted rheograms are observed, where decreasing rate curves fall below increasing curves. This response can be explained as formation of slip gap between sheared suspension and rotational cylinder due to extensive early structure development and premature stiffening^152–155^.

In contrast, the S2 (siliceous) ash mixtures, despite higher base suspension density used in tests 1.20 g/cm^3^ compared to 1.13 g/cm^3^, tolerate higher ash dosages. As shown in Fig. 10, even at 40% dosages flow curves remain within the measurable range, while 40% S1 ash dosages are out of measurement device scope. The S2 systems, while showing some fluctuations, retains an relatively stable flow and does not exhibit the severe slip gap formation and stiffening. Higher dosages require base density decrease due to lack of water and brittle solid-like behavior of mixture. However, in high S2 dosages (30–40%), some rheological instabilities, such as upturns and irregular stress oscillations, at higher dosages indicate the onset of network collapse at higher shear rates. Thus, for tested S2.O siliceous ashes the workable range of ash additives is up to ~ 40%, whereas the S1.O Ca-rich ash mixtures reach their flowability limit near ~ 30% ash load. These findings quantitatively corroborate the trends qualitatively noted earlier: S1 fine ash accelerates structural build-up to such an extent that it can induce early gelation at high dosages, whereas S2 ash mainly dilutes and disperses the clay–cement matrix, maintaining fluidity even at higher solids replacement ratios.

According to order-to-disorder transition theory, concentrated suspensions shift from ordered, layer-wise flow to chaotic collision-dominated motion under increasing shear^194–196^. Frequent particle collisions dissipate energy via friction and hinder shear-induced thinning, promoting rheological thickening. At high solid loadings, the particle cluster network is sufficiently developed that further densification is constrained, and simultaneously particle motion becomes so chaotic that shear-induced dispersion is limited; thus the system stabilize into a quasi-constant viscosity regime dominated by slip-layer formation.

#### Sodium silicate (Na_2_O_n_SiO_2_∙mH_2_O) effect on rheological properties

**Fig. 17:**
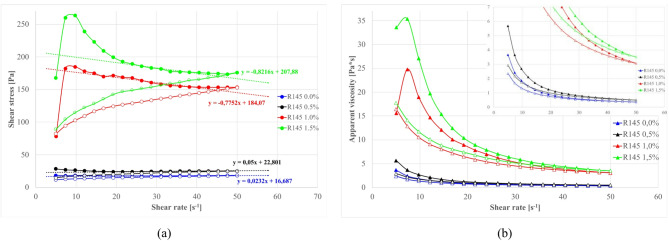
Effect of sodium silicate amount on properties of sealing suspensions as a function of shear rate: (**a**) flow curves, (**b**) apparent viscosity. Formula: base density 1.20 g/cm³, cem 5%, S1.O 5%.

**Fig. 18:**
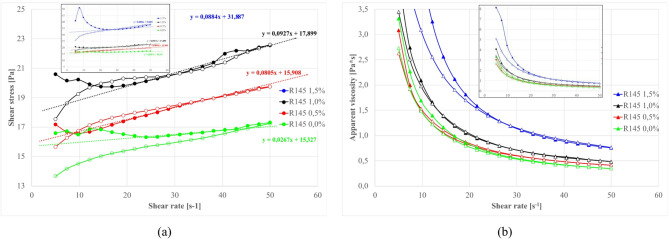
Effect of sodium silicate amount on properties of sealing suspensions as a function of shear rate: (a) flow curves, (b) apparent viscosity. Formula: base density 1.20 g/cm³, cem 5%, S2.O 5%.

The addition of a sodium silicate solution was explored as a chemical means to modify structuration. Figure 17 demonstrates the systematic influence of structure-forming additive sodium silicate content^197–202^ on rheology of sealing suspensions with S1.O ash and cement (each 5% by wt) at 1.20 g/cm³ base density. The S2.O systems response is presented in Fig. 18.

A distinct threshold phenomenon is observed with sodium silicate addition. At dosages below ~ 0.5wt %, dissolved silicate ions primarily neutralize surface charges and compress the electrical double layer, exerting only minor influence on rheology^175,199,203^. Once the concentration exceeds ~ 1.0–1.5wt %, however, both yield stress and viscosity rise abruptly—from ~ 20 Pa to > 180 Pa in the S1 system—marking a switch-like sol–gel transition. This transformation corresponds to the rapid condensation of Si–O–Si and Si–O–Al bonds catalyzed by Ca²⁺ ions released from S1 ashes and cement hydration. The resulting percolating calcium–(sodium)–aluminosilicate hydrate [C–(N)–A–S–H] network locks particles into a rigid framework and imparts pronounced shear-thinning behavior typical of alkali-activated binders^204,205^.

In contrast, the S2 (siliceous, low-Ca) suspensions display a delayed and gradual response. Appreciable viscosity increase appears only above ~ 1.5 wt % Na₂SiO₃, consistent with a slower polymerization pathway yielding Na-based aluminosilicate hydrate N–A–S–H gels^57,199,203^. The scarcity of Ca²⁺ suppresses early condensation, allowing greater silicate incorporation before gelation. Consequently, the structure that forms is softer and more reversible, exhibiting partial thixotropy and, in some cases, *negative thixotropy* when in-shear polymerization proceeds faster than structure breakdown.

The post-gel regime reveals two parallel deformation mechanisms. The newly formed C–(N)–A–S–H or N–A–S–H networks, being amorphous and polymeric, fracture irreversibly under high shear; once broken, they do not readily reform because reactive silicate species are already consumed. This accounts for the wide hysteresis loops observed in S1 mixtures. Simultaneously, a slower, reversible rebuilding occurs within the smectitic clay matrix through electrostatic edge–face and ionic edge–edge reaggregation. When the gel or ettringite frameworks dominate, this subtle clay contribution is masked by the collapse of the brittle network, whereas in their absence, mild hysteresis and structural recovery is observable. Occasionally inverted hysteresis—where descending curves exceed the ascending branch—reflects ongoing gelation (as in S2 systems) or ettringite growth (as in S1 systems) during testing, leading to apparent “over-structuring” under shear.

Overall, sodium silicate acts as a chemical trigger for sol–gel transition, but the threshold and kinetics depend strongly on the Ca/Si ratio of the ash. High-Ca S1 ashes promote rapid C–(N)–A–S–H gelation and early rigidity, while high-Si S2 ashes undergo slower N–A–S–H network formation, maintaining flowability over a wider composition range. The resulting gels differ fundamentally in mechanical character: C–(N)–A–S–H networks are stronger but brittle, whereas 3D N–A–S–H frameworks are weaker yet are more plastic and reversible.

#### Yield stress (Bingham) values

The consolidated Bingham yield stress data presented in Table 7. Base suspension density exhibits quite strong influence on yield stress values for both ash types, with S1.O systems demonstrating a steeper response gradient (19 Pa to 71 Pa) compared to S2.O systems (12 Pa to 42 Pa) across tested density ranges. This quantifies the higher sensitivity of S1 ash suspensions to base density modifications.

**Table 7:** Yield stress (Bingham) values for sealing clay-cement slurries with varying base density, cement content, fly Ash (FA) content, and sodium silicate (R145) concentrations for S1.O and S2.O formulations.

S1.O	Base denc.	Cem	FA	R145	Yeld stress	S2.O	Base denc.	Cem	FA	R145	Yeld stress
					S1.O						S2.O
	[g/cm^3^]	[%]	[%]	[%]	[Pa]		[g/cm^3^]	[%]	[%]	[%]	[Pa]
	*1*,*20*	10%	20%	0%	**71**		*1*,*22*	10%	20%	1,22 1,20 1,18	**42**
	*1*,*18*				**58**		*1*,*20*				**38**
	*1*,*13*				**19**		*1*,*18*				**12**
	1,13	10%	*40%*	0%	**n.a.**		1,20	10%	*40%*	0%	**192**
			*30%*		**440**				*30%*		**57**
			*20%*		**74**				*20%*		**38**
			*10%*		**21**				*10%*		**22**
		5%	*5%*		**17**			5%	*5%*		**16**
	1,20	5%	5%	*1*,*5%*	**208**		1,20	5%	5%	*1*,*5%*	**33**
				*1*,*0%*	**184**					*1*,*0%*	**18**
				*0*,*5%*	**23**					*0*,*5%*	**16**
				*0*,*0%*	**17**					*0*,*0%*	**15**

S1.O suspensions shows exponential yield stress increases with higher ash content, reaching 440 Pa at 30% addition—indicating intense structural build-up. In contrast, S2.O systems maintain more moderate yield stress progression, reaching only 57 Pa at equivalent 30% load. This pronounceddifference, 440 Pa versus 57 Pa (regardless lower base density 1.13 g/cm^3^ vs. 1.20 g/cm^3^), numerically confirms the significantly higher reactivity and early stage structure-forming capacity of the tested calcium-rich S1 ash and lower tolerance of such systems to solid load content.

S1.O formulations display high sensitivity to sodium silicate addition, with yield stress escalating from 17 Pa to 208 Pa. S2.O systems exhibit markedly lower sensitivity, with values rising from 15 Pa to only 33 Pa (2-fold increase) across 0% to 1.5% concentration range. This quantitative comparison provides clear evidence of the differential in between fresh-state clay-cement sealing suspensions containing S1 calcium-rich and S2 siliceous ashes.

Although the investigated suspensions exhibit pronounced yield–pseudoplastic behavior, the Bingham model was adopted here as an effective first-order approximation restricted to the quasi-steady (post-transient) region of the flow curves. This approach provides consistent, comparable parameters for assessing compositional and density-related effects across the studied systems. While the Herschel–Bulkley model is known to better capture shear-thinning behavior through the additional flow-index parameter, its use typically requires broader shear-rate ranges and more extensive fitting. Given that the present work focuses on relative trends rather than absolute rheological constants, the simpler Bingham representation was deemed sufficient and consistent with prior studies on cementitious suspensions^15,16,28,138,206^. The model thus serves here as a comparative framework rather than a full constitutive description of non-Newtonian flow.

### Cement – Ash substitution, rheological properties

Based on preliminary assessments, systematic cement substitution with fly ash fractions was conducted at constant total solid content (cem + fly ash = 10% const). This allow to obtain suspensions with a good flowability without a risk of measurement artifacts appearance and with enough system sensitivity for components impact examination. Cement-to-fly ash ratios of 10/0, 8/2, 5/5, 2/8, and 0/10 were examined with constant sodium silicate content (0.5% by total wt. of mixture) and suspensions base density (1.20 g/cm³). This enable direct comparison of substitution effects and identify optimal formulation parameters^13,188^.

#### High-Calcium (S1) Ash substitution systems

**Fig. 19:**
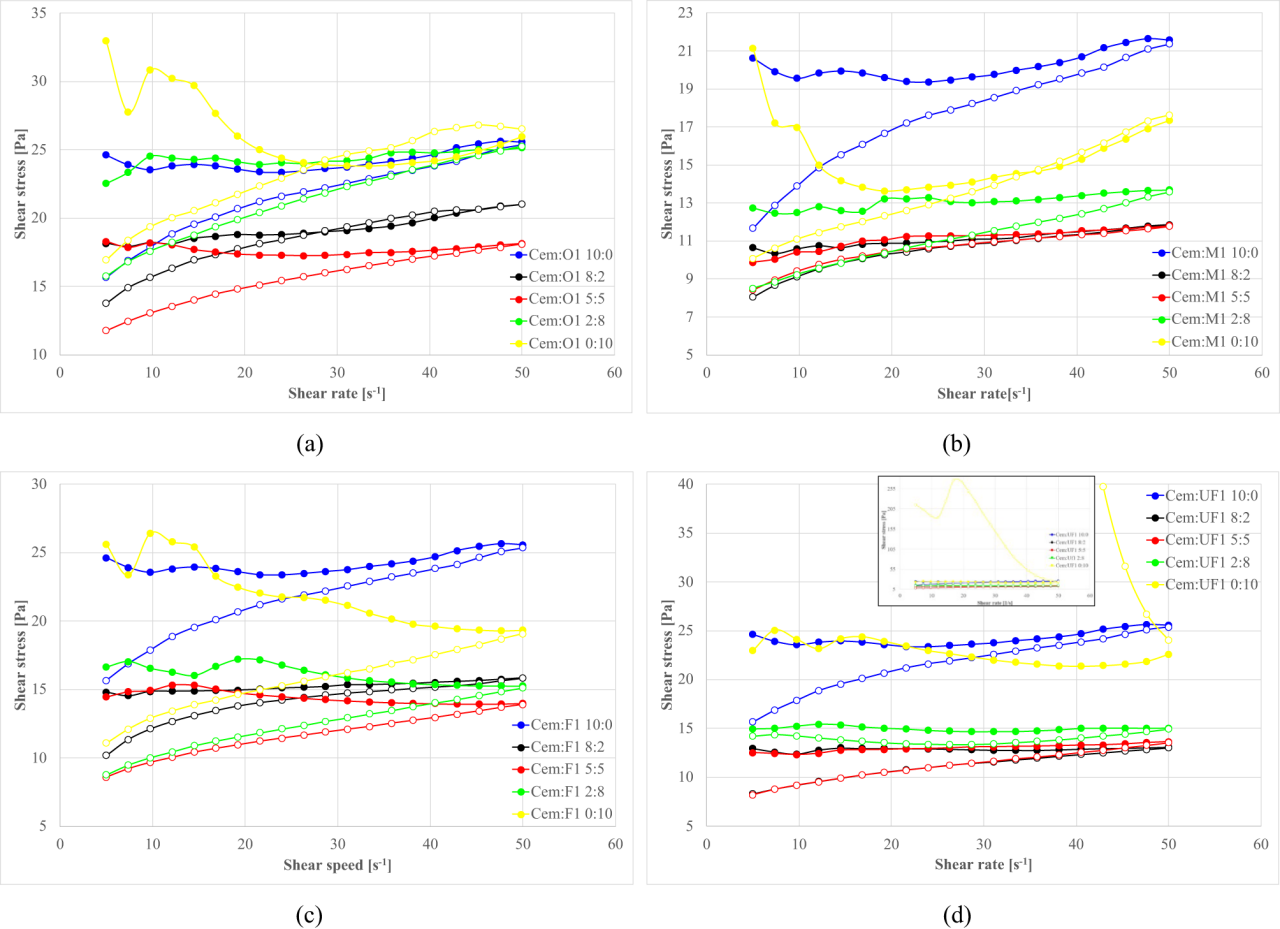
Effect of cement replacement with S1 fly ash on flow curves (**a**) S1.O, (**b**) S1.M, (**c**) S1.F, (**d**) S1.UF. Formulas: 1.20 g/cm³, load 10%*, Cem/FA 10/0, 8/2, 5/5, 2/8, 0/10, R145–0.5%. *load – the amount % by wt. of additives added to base clay-water suspension (additives cem + FA + R145).

**Fig. 20:**
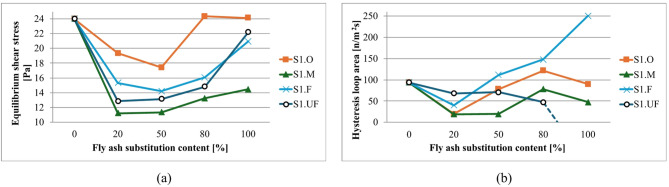
Influence of cement substitution by S1 fly ash fractions on rheological parameters of sealing suspensions (base density 1.20 g/cm³, liquid/powder 9/1 by mass, R145- 0.5%): (a) equilibrium shear stress [Pa]; (b) total hysteresis loop area [N/m²s].

Flow curves for compositions with S1 ash fractions are presented in Fig. 19, with analysis of results in terms of equilibrium shear stress and total hysteresis loop area shown in Fig. 20. Equilibrium shear stress is calculated as average shear stress for the stabilized part of the waveform (~ 20–40 s^- 1^) for upgoing curves and refers to a state where the material’s internal structure has reached a stable state after initial undergoing deformation. At this point, the rate of structure breakdown and reformation is balanced.

S1 ash addition consistently reduces equilibrium shear stress, following characteristic U-type curves with minimum values at intermediate substitution levels. The left branch (falling stress) reflects disruption of the clay–cement cluster network and optimized packing by introducing ashes to the system. At the same time, introduced ashes substitute cement in the mixture, proportionally reducing the amount of early cement hydration products^69,72,161^.

Each fraction exhibits distinct characteristics: S1.O shows highest initial stress due to larger particle sizes but achieves pronounced reduction at 5/5 substitution. Finer fractions (S1.F, S1.UF) produce consistently lower stress values with optimal performance at 5/5 ratio. Critical thresholds occur above 50% substitution where stress values begin increasing, representing transition from cement-dominated to ash-dominated systems^22,89,185,188^.

#### Power of structure destruction and rebuilding S1 Ash

**Fig. 21:**
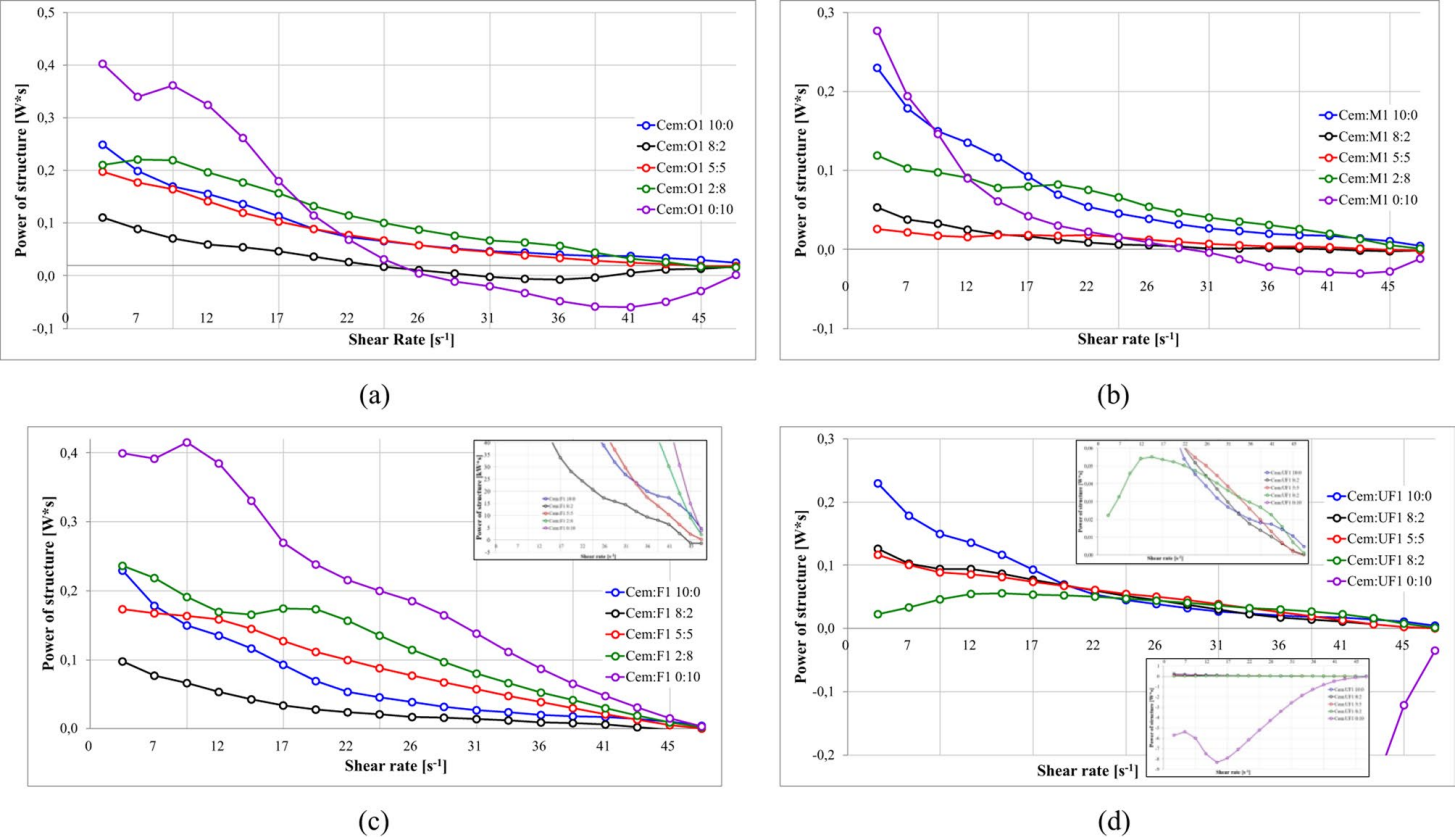
Effect of cement replacement with S1 fly ash on structure break-down/build-up power 1.20 g/cm³, load 10%, R145 0.5%, (**a**) S1.O, (**b**) S1.M, (**c**) S1.F, (**d**) S1.UF.

Figure 21 quantifies the structural dynamics across shear rates for various S1 ash fractions using power-based energy analysis, which provides superior mechanistic insights compared to simple thixotropic area calculations. Calculated as the integration of area between ascending and descending flow curves per unit time, these power curves represent instantaneous energy dissipation rates during microstructural reorganization. While thixotropic area measurements provide only cumulative energy differences, power analysis reveals real-time quantitive energy balance trends. Positive values indicate structural degradation (energy dissipation through particle network breakdown), while negative values reflect structural recovery (energy storage during particle reaggregation)^12,138,207–209^.

S1.F at 100% replacement (Cem: F1–0:10, Fig. 21.d) shows the highest positive power values, around 0.23Ws at 20s^- 1^, indicating the intense structural breakdown from extensively developed networks. S1.UF formulations resemble similar to cement-only systems at moderate substitution levels (up to 2:8), suggesting comparable to cement fly ash reactivity. However, at full replacement (0:10), S1.UF yields negative power values (up to −8Ws), indicating intense structure formation that outpaces breakdown—excessive dosage which leads to premature stiffening. ^21,161,217^

#### Silicious (S2) Ash substitution systems

**Fig. 22:**
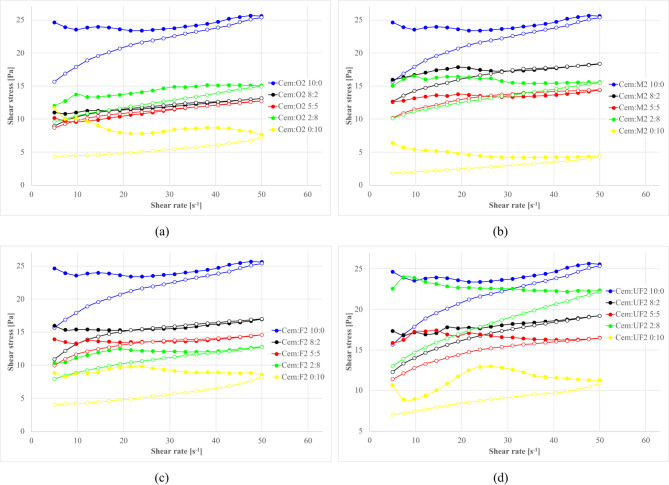
Effect of cement replacement with S2 fly ash on flow curves (**a**) S2.O, (**b**) S2.M, (**c**) S2.F, (**d**) S2.UF. Formulas: 1.20 g/cm³, load 10%, Cem/FA 10/0, 8/2, 5/5, 2/8, 0/10, R145 0.5%.

**Fig. 23:**
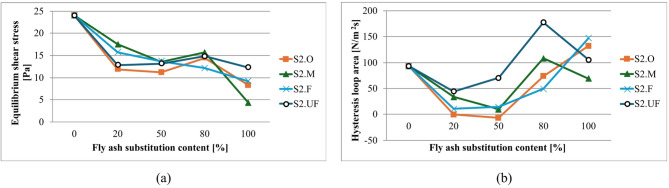
Influence of cement substitution by S2 fly ash fractions on rheological parameters of sealing suspensions (base density 1.20 g/cm³, load 10%, R145- 0.5%): (**a**) equilibrium shear stress [Pa]; (**b**) total hysteresis loop area [N/m²s].

For S2 ash systems, as illustrated in Figs. 22 and 23, the rheological behavior follows similar fundamental principles but with distinct quantitative characteristics compared to S1 ashes.

For S2 systems, equilibrium yield stress generally decreases with substitution and then plateaus from ~ 20–80%, reflecting dilution of reactive cement, disruption of the clay–cement network, and the dominance of smooth, largely inert, near-spherical particles that preserve laminar flow and suppress thixotropy^4,189,210–212^. At full substitution, fractions diverge: S2.UF continues to reduce stress (over-dispersed, weak interparticle interactions), whereas S2.O can shows a slight increase, probably due to packing/sedimentation-induced stiffness. Relative density of ashes compared to cement is lower, which results in an increase of solid particles volume and consequently disrupts clay-cement network resulting in higher thixotropy for coarser fractions (as was discussed earlier).

Hysteresis analysis reveals that even low S2 levels (20%) significantly reduce thixotropy, reflecting weaker internal networks from spherical particle morphology disrupting electrostatic clay frameworks and suppressing gel-phase formation. Higher substitution increases hysteresis for S2.O due to larger particle crowding, while finer fractions at 100% substitution shows reduced hysteresis indicating over-dispersed systems. Such dispersion can be desired in obtaining low-viscosity suspensions. Overall reduced S2 thixotropy, compared to S1 mixtures (12 Pa vs. 18 Pa for S2.O and S1.O at 5:5 subst. resp.), translates to improved workability and sustained fluidity with higher suitability for applications requiring extended open time and deep penetration^1,8,17,29,213^.

#### Power of structure destruction and rebuilding S2 Ash

**Fig. 24:**
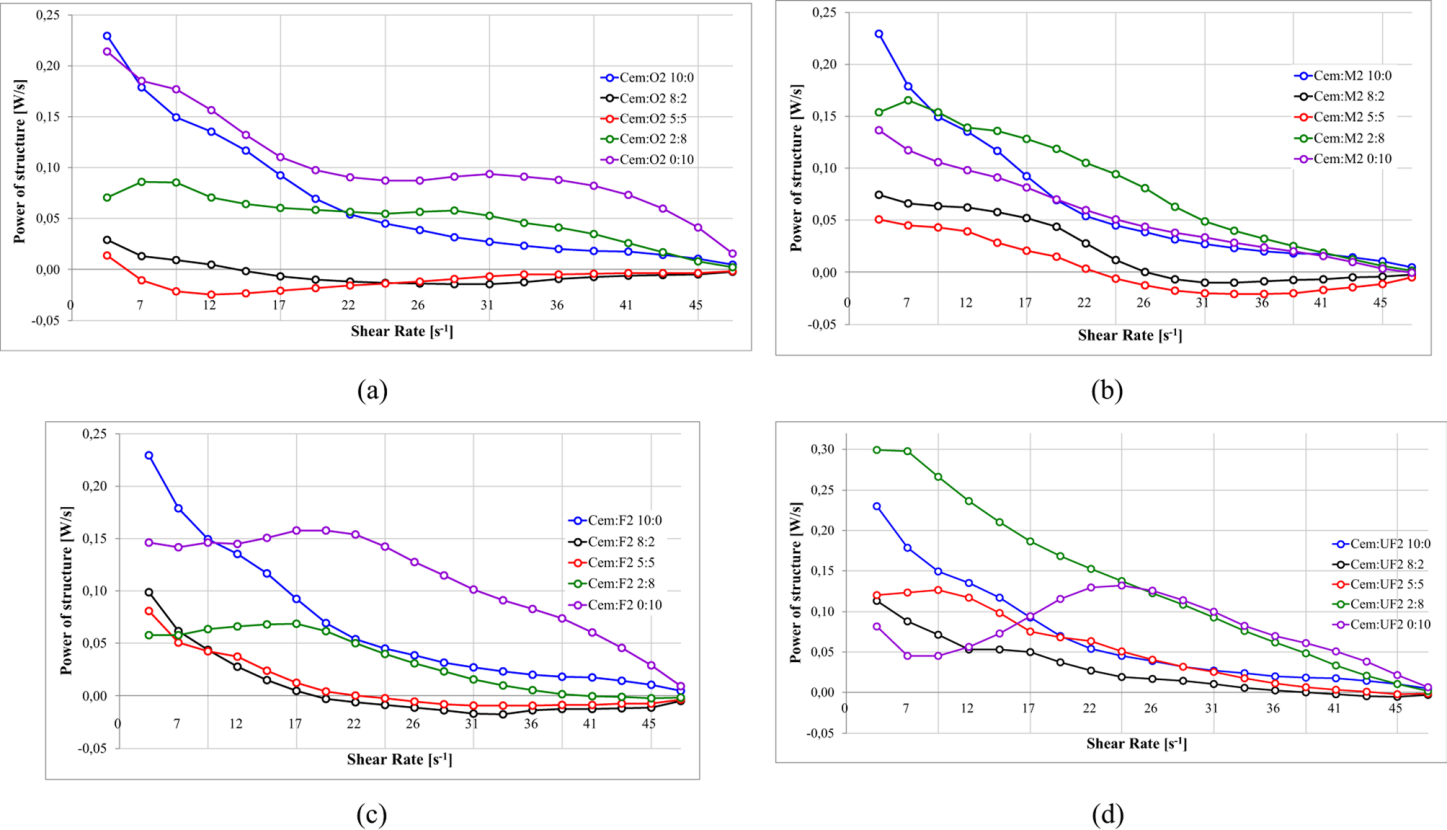
Effect of cement replacement with S2 fly ash on structure break-down/build-up power; (**a**) S2.O, (**b**) S2.M, (**c**) S2.F, (**d**) S2.UF. Formulas: 1.20 g/cm³, load 10%, R145 0.5%.

Figure 24 provides a quantitative assessment of the power of structure destruction/rebuilding as a function of shear rate for various S2 ash fractions at different substitution levels.

S2.O power curves shows systematic reductions in power with increasing shear rate, similar to S1 but with reduced magnitude, confirming weaker spatial structures in siliceous systems. S2.M formulations exhibit distinguishable pattern: moderate substitution levels (20% and 50%) produce lower destruction power than pure cement systems with negative values, suggesting structure formation during shear rate decrease. This suggests local intensifications of both structure-forming processes: hydration cased by time depended dissolution of components catalysed by intense shearing; and presence of spatial clay matrix rebuilding during diminishing flow curves^117,164,214,215^. Probably shear induced dissolution of components might dissolve spherical ash surfaces and expose roughness, however, taking into account a relatively short measurement time, this aspect may not have a large impact or rheology. SEM image analysis and surface area change be useful to indicate this effect^143,216,217^ and was not in aim of the study. High substitution levels (80–100%) overall increase power requirements, indicating flow thinning at low concentrations but contribution to structure formation and flow thickening when dominant.

S2.UF shows smallest variation across substitution levels, with nearly identical profiles to cement-only mixtures for 0–50% replacement and increases only at higher substitution levels. This consistency indicates seamless integration into cement matrix without altering structural resistance. Unlike S1.UF’s extreme negative power at high substitution, S2.UF maintains positive values, confirming absence of rapid reactive structure formation.

### Viscoelastic properties from oscillatory measurements

#### Viscoelastic properties of S1 Ash suspensions

To complement the steady-shear tests, oscillatory rheology was performed to probe the suspensions’ solid-like versus liquid-like behavior. Oscillatory rheometry provides complementary insights into the microstructure and viscoelastic characteristics that cannot be fully captured by flow curve analysis alone. These measurements enable quantification of the elastic (storage modulus G’) and viscous (loss modulus G”) components at frequency or amplitude sweep.

Frequency sweeps allows for the evaluation of how rapidly the internal structure can response to dynamic loading. This allows to determine whether materials remain elastic (G′ > G″) or transition to viscous (G″ > G′) under different frequencies and reflects the system’s resilience to vibration, pumping, or shear cycles. In contrast, amplitude sweeps (at constant frequency) assess how much force or deformation the structure can withstand before it yields^12,137,140,218^.

**Fig. 25:**
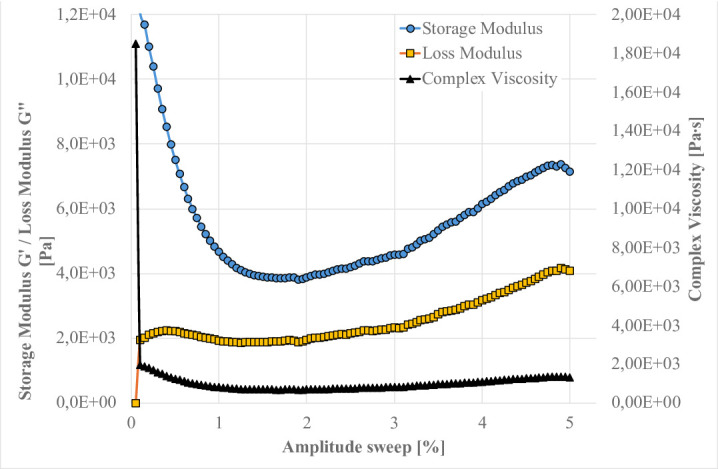
Evolution of the storage modulus (G′), loss modulus (G″), and complex viscosity as a function of strain amplitude for the Bełchatów clay–base suspension (ρ = 1.20 g/cm⁻³) containing 10.0 wt% Portland cement and 0.5 wt% sodium silicate (R145), measured at 1 Hz using a plate to plate geometry PP25 (25 mm plate).

Preliminary amplitude sweeps were first conducted to delineate the Linear Viscoelastic Region (LVR), within which both G′ and G″ remain stable in function of strain. For the present clay–cement systems, this region extends up to approximately 5% strain amplitude (Fig. 25). Beyond this point, a divergence of G′ and G″ is observed, indicating the onset of shear induced clay-network rupture. Consequently, further oscillatory measurements for clay-cement mixtures with fly ashes were performed at this 5% amplitude—just beyond the mechanical yield of the clay framework—to ensure that the response captured predominantly reflects the chemical and colloidal structuration processes (hydration and gel formation) rather than the reversible deformation of the clay skeleton. This approach enables more direct observation of the evolving geopolymeric or hydrate-based networks that govern the system’s viscoelastic stiffness^12^.

**Fig. 26:**
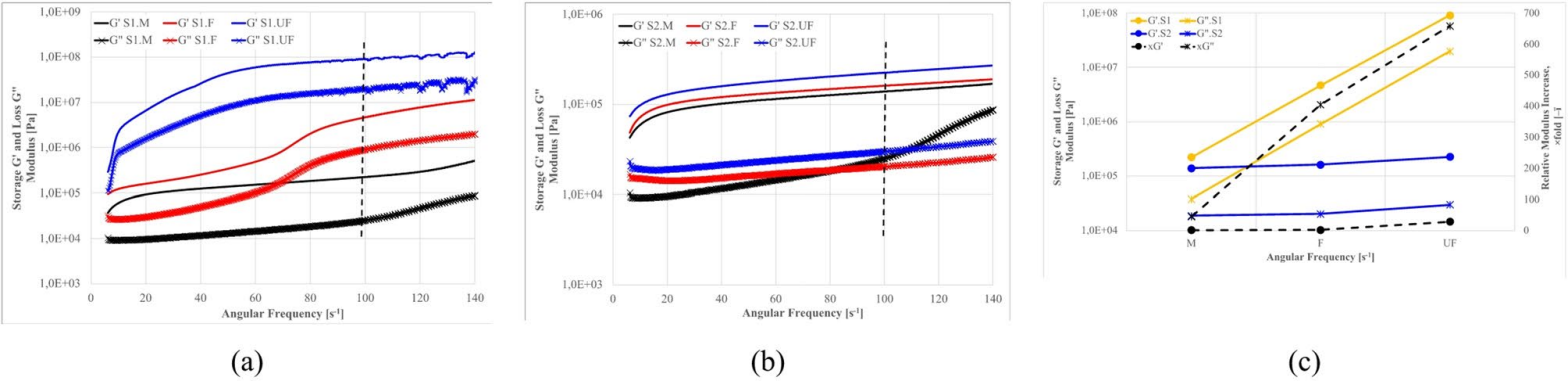
Frequency dependence of the storage (G′) and loss (G″) moduli for (**a**) S1 suspensions and (**b**) S2 suspensions prepared with medium (M), fine (F), and ultrafine (UF) ash fractions; (**c**) absolute moduli (left axis) and the modulus ratio S1/S2 for each fraction (right axis). Formulations: base density 1.18 g/cm⁻³, FA 20%, cement 10%, R 0%. The vertical dashed line in (a, b) indicates ω = 100s⁻¹ used for quantitative comparisons.

As shown in Fig. 26, all mixtures display G′ > G″ across the explored frequencies, evidencing predominantly elastic behavior. With increasing fineness, both S1 and S2 stiffen (rising G′ and G″), but the magnitude of that stiffening differs strongly between the systems. For the M fractions, S1 and S2 remain within the same order of magnitude (G′ ~10⁵ Pa; G″ ~10⁴ Pa at ω = 100 s⁻¹), indicating comparable network stiffness at the coarser scale. In the F fractions, S1 overcomes S2 by roughly one order of magnitude (G′ in the 10⁶Pa range and G″ in the 10⁵Pa range for S1 versus ~ 10⁵Pa and 10⁴Pa for S2), consistent with more effective interparticle contacts and early floc/hydrate development in S1 mixtures. The contrast becomes decisive in the UF fractions: at 100 s⁻¹, S1 reaches G′≈10⁸Pa and G″≈10⁷Pa, whereas S2 remains around 10⁵ and 10⁴Pa, respectively.

Panel (c) synthesizes this behavior by plotting the fold difference (S1:S2) at 100 s⁻¹, which grows systematically with fineness—from ~ 1.6× for M to ~ 20–40× for F, and up to ~ 400–650× for UF fractions (for both G′ and G″). Taken together, (a)–(c) shows that decreasing particle size amplifies the viscoelastic stiffness of S1 far more than S2, implying denser packing and/or higher early reactivity in S1 that accelerates network formation. This figure therefore provides the requested quantitative ranges for S2 and makes explicit the fineness-dependent divergence between the two ash systems.

**Fig. 27:**
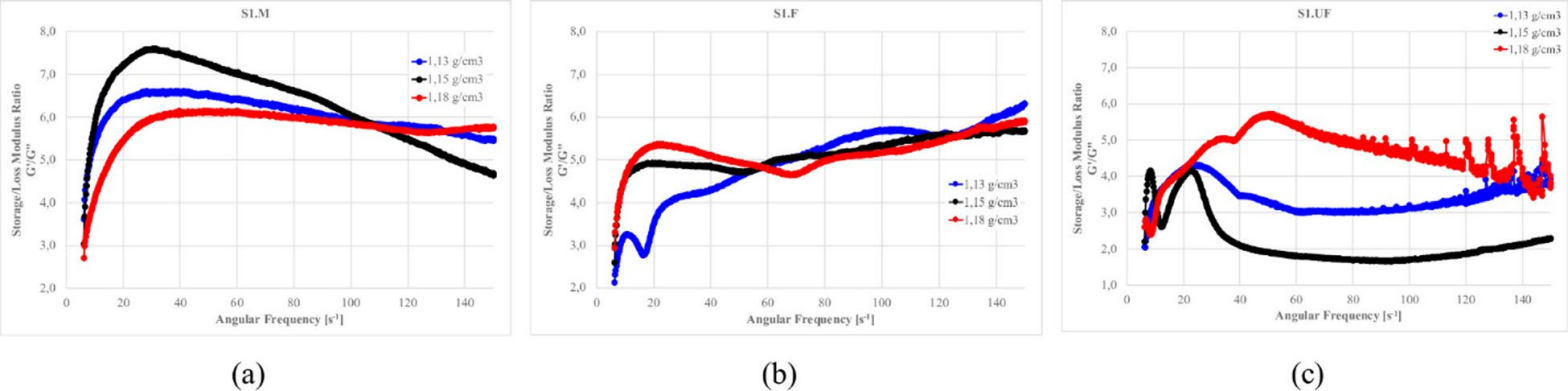
The influence of base suspension density on G’/G” module of S1 ash fractions, (**a**) M (**b**) F (**c**) UF; Formulas: FA 20% cem 10%, R 0.0%.

**Fig. 28:**
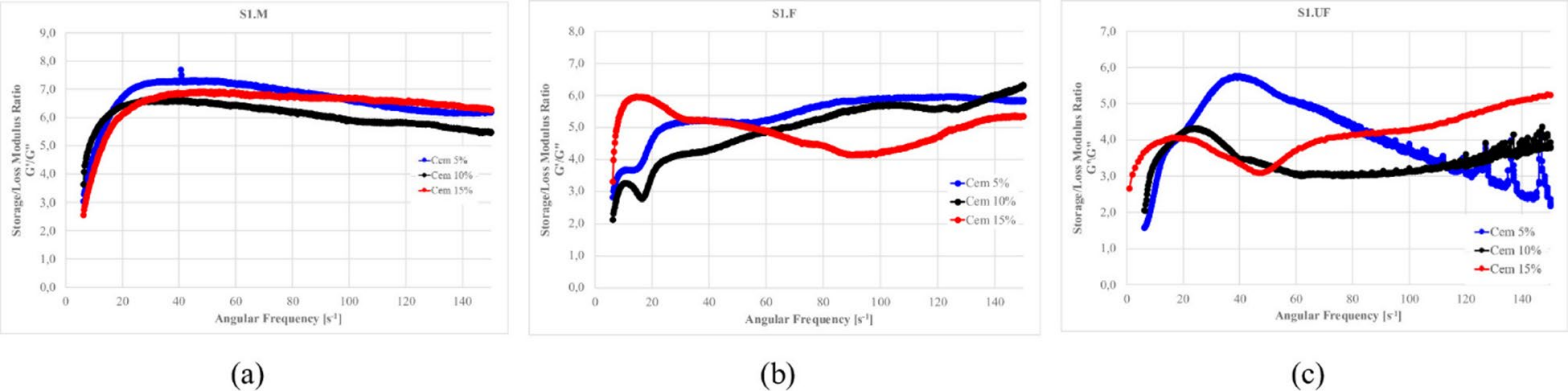
The influence cement amount on G’/G” module of S1 ash, (**a**) M (**b**) F (**c**) UF. Formulas: 1.13 g/cm^3^, FA 20%, R 0.0%.

**Fig. 29:**
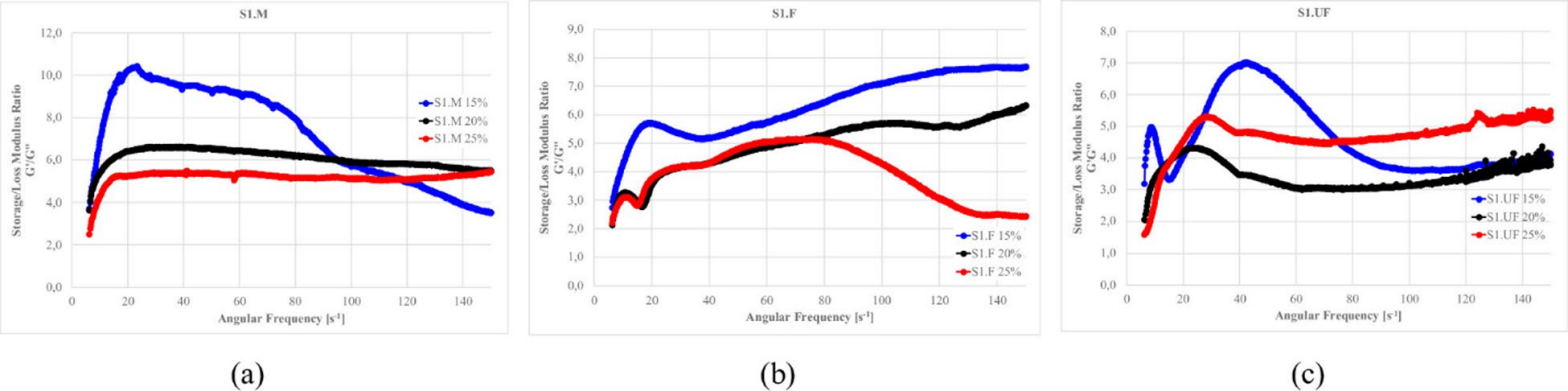
The influence S1 FA amount on G’/G” module, (**a**) S1.M (**b**) S1.F (**c**) S1.UF. Formulas: 1.13 g/cm^3^, cem 10%, R0.0%.

**Fig. 30:**
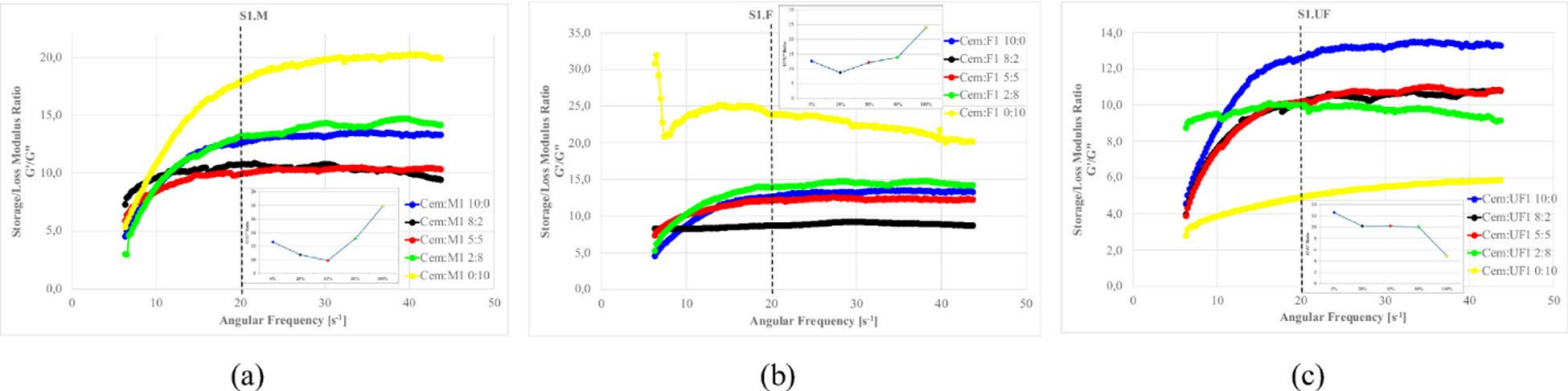
The influence of cement substitution by S1 ashes on G’/G” module: (**a**) M, (**b**) F, (**c**) UF. Formulas: 1.20 g/cm^3^, Cem/FA 10/0, 8/2, 5/5, 2/8, 0/10, R 0.5%.

Figures 27–30 presents the complex modulus (G’/G” relation) of S1 ashes, which describe the overall viscoelastic behavior of suspension as a response of oscillation shear (a sinusoidal deformation with 5% amplitude).

Oscillatory frequency sweeps revealed dominant elastic behavior across S1 formulations, with G′/G″ ratios consistently appears significantly higher that 1.0, indicating continuous internal networks with relatively high elastic contribution resistant for frequency sweeps. This structure is formed by combination of flocculated clay interactions, early cement hydration, and calcium-rich ash reactivity.

Solid loading plays a critical role in viscoelastic response. Increased suspension density, while raising apparent viscosity (as was presented at previous sections), leads to a reduced complex modulus ratio, especially in systems like S1.M. These denser suspensions appear more resistant to flow yet less capable of storing elastic deformation, indicating more rigid but less flexible networks. Notably, higher loaded structures shows greater frequency tolerance, with less sensitivity to shear-induced breakdown indicating higher floc strengths^12,22^.

At the onset of the test, a rise in the G′/G″ ratio with increasing frequency reflects shortening of time between oscillations insufficient for structural relaxation. Consequently, more of the applied shear energy is stored elastically rather than dissipated, leading to a transient stiffening effect. However, as frequency continues to increase into the medium range (10–40 rad/s), a both gradual (as in case of S1.M, Fig. 27a) and rapid (S1.UF, Fig. 27c) decline in G′/G” can be observed, suggesting that on system reorganisation capability to keep pace with imposed deformation, where in case rapid curve sweep may suggest frequency inducted elasticity threshold instead of gradual reorganisation. This reduction in elasticity implies a structure breakage and a shift toward more viscous, energy-dissipating behavior, relevant for processes involving vibration or turbulent flow, where internal structure is subjected to rapid cyclic loading^9,31,219^.

Figure 28 illustrates how increasing cement content affects the viscoelastic behavior (G′/G″) of S1-based suspensions, revealing complex interactions between calcium-induced flocculation and structural elasticity evolution that vary significantly with particle size fraction. For S1.F and S1.UF systems, increasing cement content enhances elastic contribution at higher frequencies, indicating that fine particles create synergistic networks where calcium bridging between high surface area particles (15.39–17.39.81m²/g) develops dense, interconnected structures that maintain elasticity even under rapid oscillatory loading. The mechanism involves calcium ions forming electrostatic bridges between negatively charged ash and clay surfaces, but in fine particle systems, the high particle number density and enhanced collision frequency create percolating networks with multiple stress-bearing pathways that resist frequency-induced breakdown. However, for S1.M systems, G’/G” modulus shows different behavior with frequency rise, reflecting the dual role of medium particles as both network participants and mechanical lubricants. Interestingly, cement content increase in S1.M systems (from 5% to 15%) provides enhanced frequency response stability, which can be explained by cement-induced increase of particle inter-connections allowing medium sized ash systems to obtain stability even in higher oscillation frequencies.

Increasing fly ash content (Fig. 29) generally lowers complex modulus, and related to higher level of clay-cement framework disruption by ashes and shift the response toward less elastic behaviour. However, finer ashes exhibit complex, non-monotonic responses, S1.F systems shows G′/G″ dips at intermediate frequencies (~ 13–19 rad/s), marking thresholds where structural breakdown exceeds relaxation capacity, causing initial transient fluidization followed by subsequent multiple recovery and disruption patters. The intensification of disruptions dynamics at higher frequencies (60–80 Hz, around 16–21 min after mixing) is related to ash reactivity (discussed earlier). Systems exit the linear viscoelastic region (LVR), as in case of S1.UF no longer can adequately response to applied oscillations.

Substitution-level comparisons (Fig. 30) were limited to 45 Hz (resp. to 12 min after initial mixing) to avoid measurement disturbances cased by early reactivity and to examine the influence of cement-ash substitution level on pre-reactive rheology. G′/G″ ratios follow U-shaped profiles across cement substitution levels. Up to ~ 50% replacement, systems maintain stable viscoelastic properties balancing structure formation and breakdown. At full substitution, S1.F and S1.M shows sharp G′/G″ increases reflecting network rigidification from Ca-driven flocculation. Worth noticing is S1.UF full substitution curve, which exhibits persistently lower G′/G″ compared to other curves. Taking into account high reactivity potential of S1.UF fraction, rather than weaker structuration, such elastic compound drop can be explained as over-exceeding flocculation resulting in measurement slip surfaces, similar as was discussed earlier in cylindrical system, Fig. 9b), highlighting oscillatory method limitations in highly reactive, fine-particle systems.

#### Viscoelastic properties of S2 ash suspensions

Overall, S2 ash systems exhibit comparable G′/G″ ratios, with minimal deviations in elastic network development indicating higher stability. Lacking early chemical structuration, S2 ashes influence rheology primarily through physical mechanisms. Smooth, spherical morphology promotes fluidity by minimizing interparticle friction, yielding stable profiles that resist rapid stiffening.

**Fig. 31:**
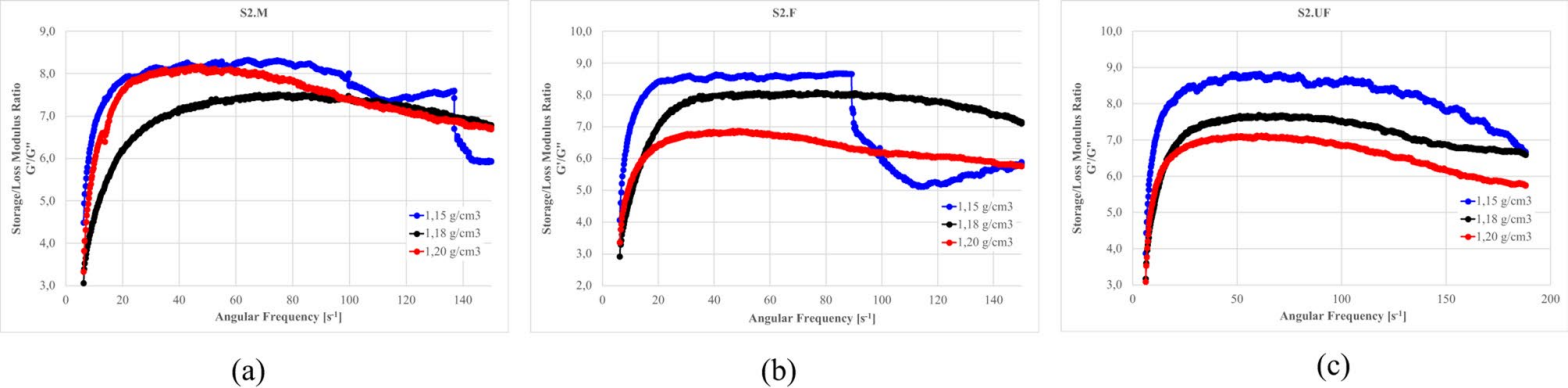
The influence of base suspension density on G’/G” module of S2 ash fractions, (**a**) M (**b**) F (**c**) UF; Formulas: FA 20% cem 10%, R 0.0%.

**Fig. 32:**
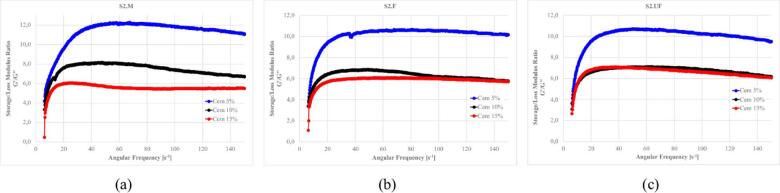
The influence cement amount on G’/G” module of S2 ash, (**a**) M (**b**) F (**c**) UF. Formulas: 1.20 g/cm^3^, FA 20%, R 0.0%.

**Fig. 33:**
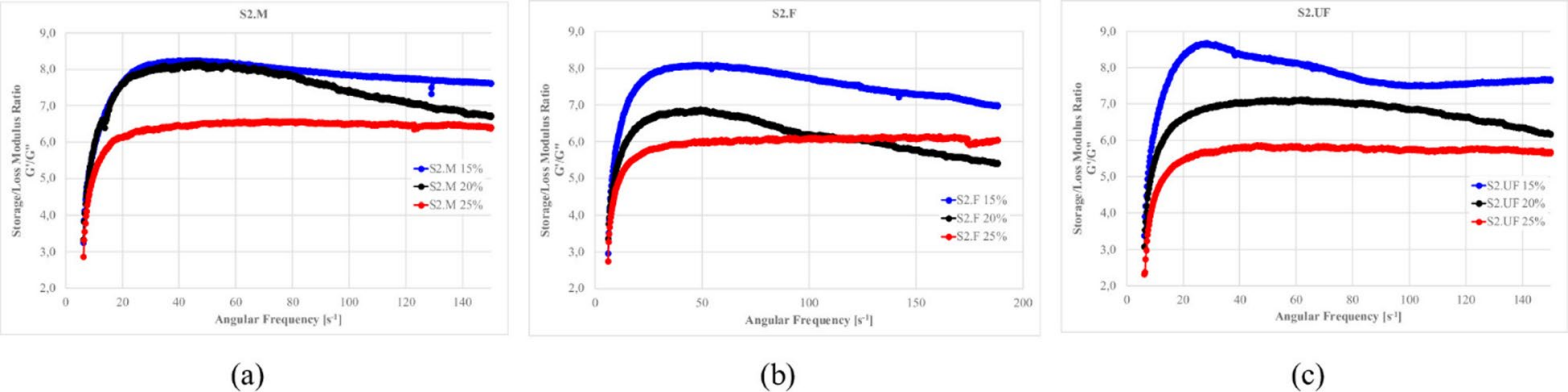
The influence S2 FA amount on G’/G” module, (**a**) S2.M (**b**) S2.F (**c**) S2.UF. Formulas: 1.20 g/cm^3^, cem 10%, R0.0%.

**Fig. 34:**
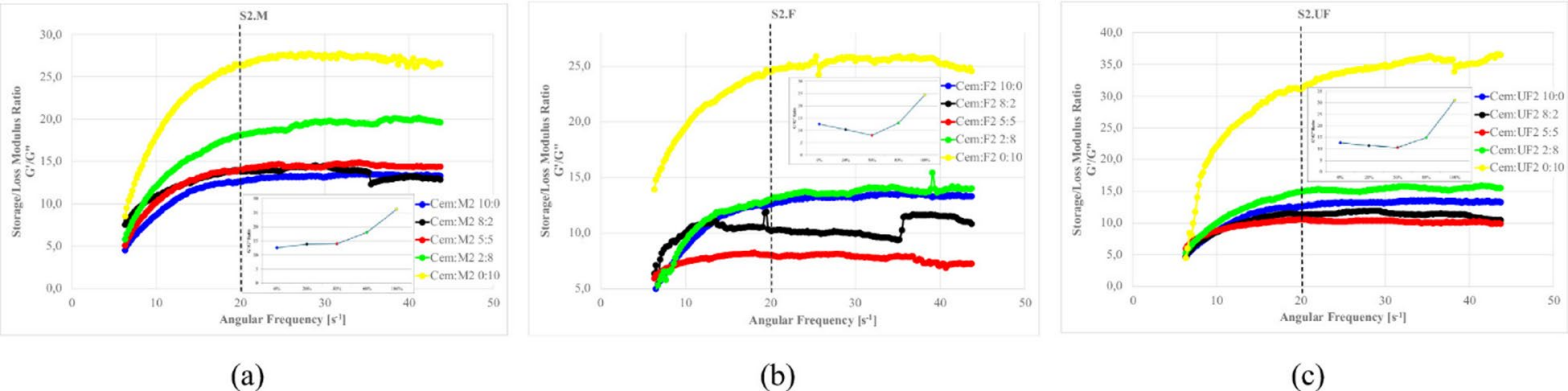
The influence of cement substitution by S2 ashes on G’/G” module: (**a**) M, (**b**) F, (**c**) UF. Formulas: 1.20 g/cm^3^, Cem/FA 10/0, 8/2, 5/5, 2/8, 0/10, R 0.5%.

Unlike S1’s dual-phase transitions, S2 suspensions shows monotonic G′/G″ behavior—ratios either decrease gradually or remain flat with frequency increase. This suggests that siliceous fly ash acts as rheological modifier rather than reactive binder.

Base density (Fig. 31) modulates viscoelastic behavior in lesser extend compared to S1 systems. Observed responses shows nearly identical G′/G″ profiles in function of tested densities, pointing on stability and similarity of systems. Lower densities shows transient deviations at higher frequencies, this may signal weak structural disruption, underscoring the inherently lower solid-phase cohesion in low density suspensions.

Figure 32 shows that increasing the cement load up to 15% lowers the complex modulus. Extra cement raises pore-solution ionic strength, creating sufficient electrolyte concentration to compress the electrical double layer around clay platelets and overcome electrostatic repulsion forces that maintain the rigid clay network structure. This ionic strength increase dismantles the original face-to-face and edge-to-face clay platelet arrangements that provide high elastic modulus through direct particle-particle contacts and van der Waals interactions. As hydration progresses, the rigid clay skeleton becomes progressively replaced by cement hydration gel networks, with appears to exhibit lower elastic component^12,215,218,220,221^. The slope of the frequency sweep remains almost unchanged, indicating comparable stability despite the reduced stiffness—i.e., the system deforms more readily across all tested frequencies once clay–clay links are replaced by C-S-H bridges.

Increasing S2 ash content (Fig. 33) systematically reduces G′/G″ values, consistent with progressive disruption of the clay–cement framework. This is attributed to the increase of smooth, inert ash grains, which lower structural rigidity and increase fluid mobility compared to pure clay-cement matrix^215,222^. Duong et al.^214^ reported the same trend for clay colloids, attributing it to the high surface charge of siliceous ash, which injects extra negative sites, intensifies Brownian motion, and keeps clay platelets dispersed rather than flocculated—hence the lower elastic contribution.

Cement substitution test results shows (Fig. 34), that even at high substitution levels (up to 80%), S2 systems exhibit G′/G″ ratios that remain lower or comparable to those of cement-only suspensions. This confirms the limited reactivity of siliceous particles over the timescales and frequencies tested, and their ability to preserve flowability without inducing premature stiffening—an advantage in applications where extended workability is critical. At 100% substitution suspensions obtain an elastic G’/G” modulus boost, probably related to the absence of cement inducted clay-clay-bridge disruption and ash low reactivity level^204,218,221^.

Notably, no sharp increases in G′/G″ are observed at high frequencies, even at full cement replacement (Fig. 34). This absence of a viscoelastic transition highlights the fundamentally physical role of S2 ashes in structure formation. Their effect arises not from gel formation or hydration, but from optimized packing, minimal surface interactions, and friction modulation—providing predictable rheological evolution across a wide range of processing conditions.

In summary, oscillatory tests support the trends deduced from steady rheology. Both ash types impart an elastic character to the fresh slurries, but Ca-rich ashes build a rigid, surge-prone network that can break or slip under stress, whereas Si-rich ashes yield softer, more resilient gels that maintain coherence under agitation. Increasing ash content or replacing too much cement tends to weaken the overall elastic scaffold in fresh state. These viscoelastic insights are important for predicting stability and segregation: a higher G′ at rest helps resist sedimentation, but if G′ is too high (and thixotropy too low), the mixture may not flow uniformly. Thus, a balance in rheology must be struck, as elaborated next.

## Implications and applicability

The rheological behaviour observed in this study shows that clay–cement slurries modified with fractionated fly ashes evolve through distinct, composition-dependent structuration pathways, and that these pathways can be directly used as design tools for levee and hydraulic-barrier applications. High-calcium S1 fractions produced a rapid two-stage viscosity rise: in the most reactive mixes, the Bingham yield stress τ₀ increased from initial values below 50 Pa to more than 200 Pa within 30–40 min, and exceeded 400 Pa for the highest S1 dosages, whereas plastic viscosity η remained in the order of 0.5–1.0 Pa·s. This pattern corresponds to an initial clay double-layer collapse followed by hydrate-induced network formation, and is mechanistically similar to the strong yield-stress growth reported for slag–cement–bentonite (SCB) walls where τ₀ typically increases several-100% in the first hour as hydration proceeds^6,11,223^. By contrast, siliceous S2 mixes with 30–40wt% ash and 10wt% cement developed much lower and more slowly increasing yield stresses, τ₀ ≈ 15–60 Pa after 30–40 min, with η ≈ 0.6–0.9 Pa·s, and maintained a quasi-linear viscosity–time profile. These values fall within the same order of magnitude as cement–bentonite trench slurries, where recommended τ₀ lies around 10–30 Pa and plastic viscosity in the range 0.012–0.078 Pa·s for good pumpability^8,224^. The important point is that our S2-based suspensions achieve comparable yield stresses despite a much higher solid volume fraction and the presence of clay, which confirms their suitability as pumpable levee slurries rather than only as laboratory pastes.

In practical terms, this means that fine and ultrafine S1 fractions behave as built-in accelerators: their rapid stiffening and high final τ₀ (often > 200 Pa within less than one hour) make them attractive for emergency applications where a slurry must quickly transition from a flowable state to a hydraulic barrier capable of blocking seepage. In the slurries studied by Cao et al.^224^, the control mix exhibited τ₀ ≈ 14 Pa and SEBS additions increased τ₀ to 23–32 Pa, still an order of magnitude lower than the most reactive S1 mixes in this work. Our S1-UF formulations therefore occupy a “rapid-plugging” niche beyond conventional cement-bentonite (CB) and slag-cement-bentonite (SCB) formulations: their yield stresses and elastic moduli are closer to those of very stiff grouts used for crack sealing than to low-strength trench backfills. At the opposite end, S2-rich systems behave as flow-preserving extenders: at 30–40wt% replacement they keep τ₀ in the 20–60 Pa window, comparable to typical cement–bentonite and grout slurries used in cut-off walls and structural grouting^9,14^. They are therefore particularly suitable where large volumes must be pumped or injected to depths of several metres without the risk of premature loss of workability.

Granulometry adds a second, independent control. In our experiments, shifting from coarse to fine or ultrafine S1 fractions more than doubled the early-age yield stress at a fixed ash content, while accelerating the time at which τ₀ exceeded 100 Pa; conversely, fine S2 fractions reduced τ₀ by roughly a factor of two compared with the S1 mixtures at equal replacement level. This is consistent with paste-scale studies by Bentz et al.^161^, who showed that changing cement and fly-ash particle size distributions can shift yield stress and viscosity by orders of magnitude and that percolation-type thresholds arise when the number density of fine reactive particles crosses a critical value. Ma et al.^225^ similarly reported that replacing cement with separated or processed fly ash decreases τ₀ and η for well-rounded, finer fractions but may increase both parameters for highly angular ash. Our data place these generic observations in a sealing-slurry context: ultrafine S1 behaves as a strong structuration accelerator producing τ₀ > 200 Pa and very high G′, suitable for fast-setting, anti-washout barriers; ultrafine S2 preserves low τ₀ and moderate G′, enabling long pumping distances and uniform filling of deep or extensive cut-offs.

The Na₂SiO₃ activation threshold introduces a third design lever. For S1-based slurries, increasing Na₂SiO₃ above about 1.0–1.5wt% (of water) produced an abrupt jump in τ₀, from values on the order of 80–120 Pa to > 200 Pa at similar hydration time, and visibly transformed the mixture from a cohesive suspension into a gel-like material. This behaviour parallels the sol–gel transitions and rapid formation of C–(N)–A–S–H networks observed in alkali-activated slag and fly-ash binders at critical activator/binder ratios^57,226^. For S1 in the present system, that threshold is operationally important: below ≈ 0.5 wt% Na₂SiO₃, activator can be used to slightly tighten the structure and reduce bleeding while still allowing pumping; above ≈ 1.0wt%, the same additive effectively converts the slurry into a fast-setting gel that is unsuitable for long-distance pumping but ideal when rapid local sealing is required (e.g., stopping concentrated leaks in levee foundations). In S2 mixtures, no such catastrophic loss of flow was observed up to 1.5wt% Na₂SiO₃; τ₀ increased moderately but remained within a pumpable range. This indicates that sodium silicate can be used as a fine-tuning tool for S2-based slurries, boosting early stiffness without sacrificing their long open time—an approach analogous to that used by Szostak at all^227^., who combined siliceous FA with C–S–H nano-admixtures to recover early mechanical performance.

Oscillatory rheology provides a direct measure of early-age structural build-up. In our tests, S1-UF systems reached storage moduli on the order of 10⁷–10⁸Pa at high frequencies, whereas S2 systems stabilized around 10⁴–10⁵ Pa—three orders of magnitude lower stiffness under comparable conditions. Such a divergence far exceeds the modest G′ increases typically observed in conventional or FA-modified cement pastes and indicates that S1-rich suspensions form a rigid, percolated skeleton almost immediately, while S2-rich systems remain comparatively soft and dissipative. Literature on cementitious slurries shows that higher early-age G′ correlates with faster network formation and improved resistance to cracking, particularly in nano-modified or slag-enhanced formulations, which stiffen rapidly through combined thixotropic and chemical contributions^228^. Recent studies demonstrates that both flocculation-driven thixotropic rebuilding and hydration-driven solidification contribute to this increase in stiffness, and that mixtures containing nano- or mineral admixtures tend to exhibit accelerated G′ growth due to higher surface area, enhanced nucleation, or stronger interparticle attractions^229–232^. Within this framework, our S1-UF mixtures align with high-G′ sealing slurries designed for rapid structural robustness, whereas S2 mixtures fall into a low-G′ mixtures, that tolerates vibration and long pumping distances without structure breackage. The observed G′/G″ evolution thus provides a practical criterion for mixture selection: rapidly rising G′ for fast sealing performance and stable, moderate G′ for vibration-tolerant placement.

Overall, the combination of obtained data with literature benchmarks shows that the three main levers identified here—ash chemistry (S1 vs. S2), particle fineness, and Na₂SiO₃ dosage—allow designers to move deliberately within a rheological design space that is already well recognised in CB/SCB and FA-modified systems, but has not previously been mapped for clay–cement–fly ash levee slurries, especially in particle fineness obtained by aerodynamic means. In that sense, the present work confirm qualitative trends and it provides quantified boundaries (τ₀ levels, η ranges, G′ magnitudes, activator thresholds) that can be directly used to formulate mixtures for: (i) fast-plugging of local seepage paths, (ii) long-distance pumping of large slurry volumes, and (iii) low-CO₂ cut-off walls where high cement replacement is required without sacrificing rheological performance.

## Conclusions

This work shows that the rheological behaviour of clay–cement sealing slurries is highly sensitive to the chemistry and particle-size distribution of added fly ashes, and that these effects can be used strategically to control early structuration and workability. High-calcium fractions promote rapid network formation and early stiffness, while siliceous fractions disperse the clay–cement framework and prolong fluidity. Particle fineness amplifies these tendencies, acting as an effective lever for accelerating or delaying structural build-up.

The study also demonstrates that cement can be substantially replaced with fly ash without compromising slurry stability, and that sodium-silicate activation introduces a clear transition between flowable and rapidly gelling regimes. Viscoelastic measurements further highlight the fundamentally different microstructures formed by calcium-rich and siliceous ashes, providing an additional tool for predicting field behaviour.

Together, these findings establish a coherent framework linking ash characteristics with rheological response and application performance. They shows that fractionated fly ashes enable purposeful tailoring of slurry behaviour—from highly flowable mixtures suited for deep, large-scale injection to fast-setting compositions intended for rapid sealing—while supporting more sustainable, lower-cement formulations.

## Data Availability

The datasets generated during and/or analysed during the current study are available from the corresponding author on reasonable request.

## References

[1] Huang, X. et al. Use of self-hardening slurry for trench cutoff wall: A review. *Constr. Build. Mater.* **286**, 122959 (2021).

[2] Skutnik, Z., Bajda, M. & Lech, M. The selection of sealing technologies of the subsoil and hydrotechnical structures and quality assurance. *Open. Eng.* **9**, 420–427 (2019).

[3] Bourg, I. C., Ajo-Franklin, J. B. & Clay Water, and salt: controls on the permeability of Fine-Grained sedimentary rocks. *Acc. Chem. Res.* **50**, 2067–2074 (2017). 28862427 10.1021/acs.accounts.7b00261

[4] Jefferis, S. Cement-Bentonite Slurry Systems. in *Grouting and Deep Mixing 2012* 1–24American Society of Civil Engineers, Reston, VA, (2012). 10.1061/9780784412350.0001

[5] Kipko, E. R. & Połozow, J. A. Kompleksnyj metod tamponaza pri stroitelstwie szacht. *Nedra, Moskwa* (1984).

[6] Ryan, C. R. & Day, S. R. Soil-Cement-Bentonite Slurry Walls. in *Deep Foundations 2002* 713–727American Society of Civil Engineers, Reston, VA, (2002). 10.1061/40601(256)51

[7] Inazumi, S., Tazuke, K. & Kashima, S. Time-Dependent rheological behavior and MPS simulation of Cement–Bentonite slurries with hydration accelerators for borehole backfilling applications. *J. Compos. Sci.* **9**, 361 (2025).

[8] Ryan, C., Ruffing, D. & Evans, J. C. Soil Bentonite Slurry Trench Cutoff Walls: History, Design, and Construction Practices. in *Geo-Congress 2022* 89–99American Society of Civil Engineers, Reston, VA, (2022). 10.1061/9780784484050.010

[9] Njock, P. G. A., Chen, J., Modoni, G., Arulrajah, A. & Kim Y.-H. A review of jet grouting practice and development. *Arab. J. Geosci.* **11**, 459 (2018).

[10] Yang, Z., Zhang, Y., Zhu, Y., Li, Y. & Fu, R. The Strength, Permeability, and microstructure of Cement–Bentonite Cut-Off walls enhanced by polypropylene fiber. *Sustainability* **17**, 3656 (2025).

[11] Jefferis, S. A. Bentonite-cement slurries for hydraulic cut-offs. *International Conference Soil Mechanics and Foundation Engineering* 1, 435–440 (1981).

[12] Izak, P. Reologia w ceramice. *Wydawnictwa AGH: Kraków, Poland* (2015).

[13] Stempkowska, A., Wójcik, Ł., Ostrowski, K. A. & Gawenda, T. Low-Energy Clay–Cement slurries find application as waterproofing membranes for limiting the migration of Contaminants—Case studies in Poland. *Energies (Basel)*. **16**, 230 (2022).

[14] Benyounes, K. Rheological behavior of cement-based Grout with Algerian bentonite. *SN Appl. Sci.* **1**, 1037 (2019).

[15] Onyelowe, K. C. & Kontoni, D. P. N. A critical review of rheological models in self-compacting concrete for sustainable structures. *Sci. Rep.* **13**, 21296 (2023). 38042887 10.1038/s41598-023-48673-6 PMC10693590

[16] Tao, C., Kutchko, B. G., Rosenbaum, E. & Massoudi, M. A. Review of rheological modeling of cement slurry in oil well applications. *Energies (Basel)*. **13**, 570 (2020).

[17] Jiang, H., Fall, M., Yilmaz, E., Li, Y. & Yang, L. Effect of mineral admixtures on flow properties of fresh cemented paste backfill: assessment of time dependency and Thixotropy. *Powder Technol.* **372**, 258–266 (2020).

[18] Mesboua, N., Benyounes, K. & Benmounah, A. Study of the impact of bentonite on the physico-mechanical and flow properties of cement Grout. *Cogent Eng.* **5**, 1446252 (2018).

[19] Zhou, H., Zhang, Y., Zhu, W., Zhong, Q. & Huang, X. Optimisation of synchronous grouting mix ratio for shield tunnels. *Appl. Sci.* **14**, 4098 (2024).

[20] Nachbaur, L., Mutin, J. C., Nonat, A. & Choplin, L. Dynamic mode rheology of cement and tricalcium silicate pastes from mixing to setting. *Cem. Concr Res.* **31**, 183–192 (2001).

[21] Roussel, N. A Thixotropy model for fresh fluid concretes: Theory, validation and applications. *Cem. Concr Res.* **36**, 1797–1806 (2006).

[22] Tregger, N. A., Pakula, M. E. & Shah, S. P. Influence of clays on the rheology of cement pastes. *Cem. Concr Res.* **40**, 384–391 (2010).

[23] Perrot, A., Rangeard, D. & Pierre, A. Structural built-up of cement-based materials used for 3D-printing extrusion techniques. *Mater. Struct.* **49**, 1213–1220 (2016).

[24] Bentz, D. P. A review of early-age properties of cement-based materials. *Cem. Concr Res.* **38**, 196–204 (2008).

[25] Mostafa, A. M. & Yahia, A. New approach to assess build-up of cement-based suspensions. *Cem. Concr Res.* **85**, 174–182 (2016).

[26] Shakeel, A., Kirichek, A. & Chassagne, C. Rheology and yielding transitions in mixed kaolinite/bentonite suspensions. *Appl. Clay Sci.* **211**, 106206 (2021).

[27] Roussel, N., Lemaître, A., Flatt, R. J. & Coussot, P. Steady state flow of cement suspensions: A micromechanical state of the Art. *Cem. Concr Res.* **40**, 77–84 (2010).

[28] Zhang, C. et al. Rheological properties of cement-based slurry and evaluation of rheological model: influence of particle size and shape. *Constr. Build. Mater.* **406**, 133498 (2023).

[29] Han, F., Pu, S., Zhou, Y., Zhang, H. & Zhang, Z. Effect of ultrafine mineral admixtures on the rheological properties of fresh cement paste: A review. *J. Building Eng.* **51**, 104313 (2022).

[30] Kaci, A., Chaouche, M. & Andréani, P. A. Influence of bentonite clay on the rheological behaviour of fresh mortars. *Cem. Concr Res.* **41**, 373–379 (2011).

[31] Tobias, S. et al. Soil sealing and unsealing: state of the Art and examples. *Land. Degrad. Dev.* **29**, 2015–2024 (2018).

[32] Juenger, M. C. G., Winnefeld, F., Provis, J. L. & Ideker, J. H. Advances in alternative cementitious binders. *Cem. Concr Res.* **41**, 1232–1243 (2011).

[33] Van den Heede, P. & De Belie, N. Environmental impact and life cycle assessment (LCA) of traditional and ‘green’ concretes: literature review and theoretical calculations. *Cem. Concr Compos.* **34**, 431–442 (2012).

[34] Ashraf, M. et al. Developing a sustainable concrete incorporating bentonite clay and silica fume: mechanical and durability performance. *J. Clean. Prod.* **337**, 130315 (2022).

[35] William, C. E., Akobo, Z. S., Ngekpe, B. E. & I. & Effect of Metakaolin as a partial replacement for cement on the compressive strength of high strength concrete at varying Water/Binder ratios. *Int. J. Civil Eng.* **6**, 1–6 (2019).

[36] Gil, D. M. & Golewski, G. L. Potential of siliceous fly ash and silica fume as a substitute for binder in cementitious concretes. *E3S Web of Conferences* 49, 00030 (2018).

[37] Robalo, K., Costa, H., do Carmo, R. & Júlio, E. Enhanced mechanical and durability performances of low cement concrete with natural Pozzolan addition. *J. Adv. Concr. Technol.* **19**, 519–535 (2021).

[38] Wilińska, I., Pacewska, B. & Ostrowski, A. Investigation of different ways of activation of fly ash–cement mixtures. *J. Therm. Anal. Calorim.* **138**, 4203–4213 (2019).

[39] Komljenović, M., Petrašinović-Stojkanović, L., Baščarević, Z., Jovanović, N. & Rosić, A. Fly Ash as the potential Raw mixture component for Portland cement clinker synthesis. *J. Therm. Anal. Calorim.* **96**, 363–368 (2009).

[40] Ramjan, S. et al. Influence of cement replacement with fly Ash and ground sand with different fineness on Alkali-Silica reaction of mortar. *Materials* **14**, 1528 (2021). 33804759 10.3390/ma14061528 PMC8004033

[41] Devarangadi, M. & Uma Shankar, M. Effect on engineering properties of ground granulated blast furnace slag admixed with laterite soil, cement and bentonite mixtures as a liner in landfill. *J. Clean. Prod.* **329**, 129757 (2021).

[42] Ahmad, J. et al. A comprehensive review on the ground granulated blast furnace slag (GGBS) in concrete production. *Sustainability* **14**, 8783 (2022).

[43] Mollamahmutoğlu, M. & Avcı, E. Engineering properties of Slag-Based superfine Cement-Stabilized clayey soil. *ACI Mater. J* **115**, (2018).

[44] Alam, M. T., Dai, B., Wu, X., Hoadley, A. & Zhang, L. A critical review of Ash slagging mechanisms and viscosity measurement for low-rank coal and bio-slags. *Front. Energy*. **15**, 46–67 (2021).

[45] Vasiliev, V. V. & Vasileva, E. A. Pozzolanic activity of fly Ash. *Silic. Indus.* **68**, 111–117 (2016).

[46] Golewski, G. L. The role of pozzolanic activity of siliceous fly Ash in the formation of the structure of sustainable cementitious composites. *Sustainable Chem.* **3**, 520–534 (2022).

[47] Hollanders, S., Adriaens, R., Skibsted, J., Cizer, Ö. & Elsen, J. Pozzolanic reactivity of pure calcined clays. *Appl. Clay Sci.* **132–133**, 552–560 (2016).

[48] Almenares, R. S. et al. Industrial calcination of kaolinitic clays to make reactive Pozzolans. *Case Stud. Constr. Mater.* **6**, 225–232 (2017).

[49] Park, C. K., Noh, M. H. & Park, T. H. Rheological properties of cementitious materials containing mineral admixtures. *Cem. Concr Res.* **35**, 842–849 (2005).

[50] Chen, H. J., Chang, H. L., Tang, C. W. & Yang, T. Y. Application of biomineralization technology to Self-Healing of Fiber-Reinforced lightweight concrete after exposure to high temperatures. *Materials* **15**, 7796 (2022). 36363387 10.3390/ma15217796 PMC9657245

[51] Awang, H., Ahmad, M. H., & Al-Mulali, M. Z. Influence of Kenaf and polypropylene fibers on mechanical and durability properties of fibre reinforced lightweight foamed concrete. *J. Eng. Sci. Technol.* **10** (4), 496–508 (2015).

[52] Khan, M., Cao, M. & Ali, M. Cracking behaviour and constitutive modelling of hybrid fibre reinforced concrete. *J. Building Eng.* **30**, 101272 (2020).

[53] Cui, W. et al. Fiber-reinforced viscoelastomers show extraordinary crack resistance that exceeds metals. *Adv. Mater.* **32** (31), 1907180. 10.1002/adma.201907180 (2020). 10.1002/adma.201907180 32583491

[54] Sun, P. et al. Rheological and mechanical properties of Bentonite–Cement paste reinforced with basalt fibers. *Materials* **16**, 3226 (2023). 37110062 10.3390/ma16083226 PMC10146701

[55] Li, Y., Shen, J., Lin, H. & Li, Y. Optimization design for alkali-activated slag-fly Ash geopolymer concrete based on artificial intelligence considering compressive strength, cost, and carbon emission. *J. Building Eng.* **75**, 106929 (2023).

[56] Salvador, R. P., Cavalaro, S. H. P., Segura, I., Figueiredo, A. D. & Pérez, J. Early age hydration of cement pastes with alkaline and alkali-free accelerators for sprayed concrete. *Constr. Build. Mater.* **111**, 386–398 (2016).

[57] Palomo, A., Grutzeck, M. W. & Blanco, M. T. Alkali-activated fly ashes. *Cem. Concr Res.* **29**, 1323–1329 (1999).

[58] Gismera, S., Alonso, M. M., Palacios, M. & Puertas, F. Rheology of Alkali-Activated mortars: influence of particle size and nature of aggregates. *Minerals* **10**, 726 (2020).

[59] Yi, Y., Li, C. & Songyu Liu. Alkali-activated ground-granulated blast furnace slag for stabilization of marine soft clay. *J. Mater. Civil Eng.* **27** (4), 04014146 (2015).

[60] Komljenović, M., Baščarević, Z. & Bradić, V. Mechanical and microstructural properties of alkali-activated fly Ash geopolymers. *J. Hazard. Mater.* **181**, 35–42 (2010). 20554110 10.1016/j.jhazmat.2010.04.064

[61] Palacios, M., Alonso, M. M., Varga, C. & Puertas, F. Influence of the alkaline solution and temperature on the rheology and reactivity of alkali-activated fly Ash pastes. *Cem. Concr Compos.* **95**, 277–284 (2019).

[62] Kala, K. & Subramaniam, K. V. L. Alkali-activated fly ash-blast furnace slag blend rheology: evaluation of yield and Maxwell responses. *Clean. Eng. Technol.* **6**, 100398 (2022).

[63] Alnahhal, M. F., Kim, T. & Hajimohammadi, A. Evolution of flow properties, plastic viscosity, and yield stress of alkali-activated fly ash/slag pastes. *RILEM Tech. Lett.* **5**, 141–149 (2020).

[64] Ahmaruzzaman, M. A review on the utilization of fly ash. *Progr. Energy Combust. Sci.* **36**, 327–363. 10.1016/j.pecs.2009.11.003 (2010).

[65] Khairul Nizar, H. & Kamarudin Mohd sobri Idris. Pysical, chemical & mineralogical properties of Fly-ash. *J. Nuclear Relat. Technol.* **4**, 47–51 (2007).

[66] Gollakota, A. R. K., Volli, V. & Shu, C. M. Progressive utilisation prospects of coal fly ash: A review. *Sci. Total Environ.* **672**, 951–989 (2019). 30981170 10.1016/j.scitotenv.2019.03.337

[67] Bhatt, A. et al. Physical, chemical, and geotechnical properties of coal fly ash: A global review. *Case Stud. Constr. Mater.* **11**, e00263 (2019).

[68] Das, S. K. Yudhbir. Geotechnical characterization of some Indian fly ashes. *J. Mater. Civ. Eng.* **17**, 544–552 (2005).

[69] Yang, T. et al. Effect of fly Ash microsphere on the rheology and microstructure of alkali-activated fly Ash/slag pastes. *Cem. Concr Res.* **109**, 198–207 (2018).

[70] Bicer, A. Effect of fly Ash particle size on thermal and mechanical properties of fly Ash-cement composites. *Therm. Sci. Eng. Progress*. **8**, 78–82 (2018).

[71] Das, D. et al. Stabilization and rheological behavior of fly Ash–Water slurry using a natural dispersant in pipeline transportation. *ACS Omega*. **4**, 21604–21611 (2019). 31867557 10.1021/acsomega.9b03477 PMC6921645

[72] Lee, S. H., Kim, H. J., Sakai, E. & Daimon, M. Effect of particle size distribution of fly ash–cement system on the fluidity of cement pastes. *Cem. Concr Res.* **33**, 763–768 (2003).

[73] Aigbe, U. O. et al. Fly ash-based adsorbent for adsorption of heavy metals and dyes from aqueous solution: a review. *J. Mater. Res. Technol.* **14**, 2751–2774 (2021).

[74] Lupu, C., Jackson, K. L., Bard, S., Barron, A. R. & Water Acid, and calcium carbonate pretreatment of fly ash: the effect on setting of Cement – fly Ash mixtures. *Ind. Eng. Chem. Res.* **46**, 8018–8025 (2007).

[75] Fernández-Jiménez, A., Palomo, A., Pastor, J. Y. & Martín, A. New cementitious materials based on Alkali‐Activated fly ash: performance at high temperatures. *J. Am. Ceram. Soc.* **91**, 3308–3314 (2008).

[76] MCCARTHY, M. & DHIR, R. Development of high volume fly Ash cements for use in concrete construction. *Fuel* **84**, 1423–1432 (2005).

[77] Li, G. et al. Fly Ash application as supplementary cementitious material: A review. *Materials* **15**, 2664 (2022). 35407996 10.3390/ma15072664 PMC9000507

[78] Shehata, M. H., Thomas, M. D. A. & Bleszynski, R. F. The effects of fly Ash composition on the chemistry of pore solution in hydrated cement pastes. *Cem. Concr Res.* **29**, 1915–1920 (1999).

[79] Chindaprasirt, P., Homwuttiwong, S. & Sirivivatnanon, V. Influence of fly Ash fineness on strength, drying shrinkage and sulfate resistance of blended cement mortar. *Cem. Concr Res.* **34**, 1087–1092 (2004).

[80] Kasaniya, M., Thomas, M. D. A. & Moffatt, E. G. Pozzolanic reactivity of natural pozzolans, ground glasses and coal bottom ashes and implication of their incorporation on the chloride permeability of concrete. *Cem. Concr Res.* **139**, 106259 (2021).

[81] Wattimena, O. K. & Hardjito, D. Antoni A review on the effect of fly Ash characteristics and their variations on the synthesis of fly Ash based geopolymer. in **020041** (2017). 10.1063/1.5003524

[82] WARD, C. & FRENCH, D. Determination of glass content and Estimation of glass composition in fly Ash using quantitative X-ray diffractometry. *Fuel* **85**, 2268–2277 (2006).

[83] Aughenbaugh, K. L., Stutzman, P. & Juenger, M. C. G. Identifying glass compositions in fly Ash. *Front. Mater.* **3**, 1–10. 10.3389/fmats.2016.00001 (2016).

[84] Vassilev, S. V. & Vassileva, C. G. A new approach for the classification of coal fly ashes based on their origin, composition, properties, and behaviour. *Fuel* **86**, 1490–1512 (2007).

[85] Wang, Y. L., Cui, S. P., Tian, G. P., Lan, M. Z. & Wang, Z. H. Effect of fly Ash composition and structure on the formation of cement clinker. *Key Eng. Mater.* **680**, 429–434 (2016).

[86] Cho, Y. K., Jung, S. H. & Choi, Y. C. Effects of chemical composition of fly Ash on compressive strength of fly Ash cement mortar. *Constr. Build. Mater.* **204**, 255–264 (2019).

[87] Vassilev, S. V., Menendez, R., Alvarez, D., Diaz-Somoano, M. & Martinez-Tarazona, M. R. Phase-mineral and chemical composition of coal fly ashes as a basis for their multicomponent utilization. 1. Characterization of feed coals and fly ashes☆. *Fuel* **82**, 1793–1811 (2003).

[88] Li, Z., Xu, G. & Shi, X. Reactivity of coal fly Ash used in cementitious binder systems: A state-of-the-art overview. *Fuel* **301**, 121031 (2021).

[89] Ferraris, C. F., Obla, K. H. & Hill, R. The influence of mineral admixtures on the rheology of cement paste and concrete. *Cem. Concr Res.* **31**, 245–255 (2001).

[90] Gesoğlu, M., Güneyisi, E., Kocabağ, M. E., Bayram, V. & Mermerdaş, K. Fresh and hardened characteristics of self compacting concretes made with combined use of marble powder, limestone filler, and fly Ash. *Constr. Build. Mater.* **37**, 160–170 (2012).

[91] Hemalatha, T. & Ramaswamy, A. A review on fly Ash characteristics – Towards promoting high volume utilization in developing sustainable concrete. *J. Clean. Prod.* **147**, 546–559 (2017).

[92] Diamond, S. On the glass present in low-calcium and in high-calcium flyashes. *Cem. Concr Res.* **13**, 459–464 (1983).

[93] Hooton, R., Tishmack, J., Olek, J. & Diamond, S. Characterization of High-Calcium fly ashes and their potential influence on ettringite formation in cementitious systems. *Cem. Concrete Aggregates*. **21**, 82 (1999).

[94] Yao, Z. T. et al. A comprehensive review on the applications of coal fly Ash. *Earth Sci. Rev.* **141**, 105–121 (2015).

[95] Kelechi, S. E. et al. A comprehensive review on coal fly Ash and its application in the construction industry. *Cogent Eng.* **9** (1), 1–26. 10.1080/23311916.2022.2114201 (2022).

[96] Chancey, R. T., Stutzman, P., Juenger, M. C. G. & Fowler, D. W. Comprehensive phase characterization of crystalline and amorphous phases of a class F fly Ash. *Cem. Concr Res.* **40**, 146–156 (2010).

[97] Thomas, M. Optimizing the use of fly Ash in concrete. *Portl. Cem. Ass.* **5420**, 1–26 (2007).

[98] McCarthy, M. J., Zheng, L., Dhir, R. K. & Tella, G. Dry-processing of long-term wet-stored fly Ash for use as an addition in concrete. *Cem. Concr Compos.* **92**, 205–215 (2018).

[99] Lv, B. et al. Separation of unburned carbon from coal fly ash: Pre-classification in liquid–solid fluidized beds and subsequent flotation. *Process Saf. Environ. Prot.* **165**, 408–419 (2022).

[100] Petrus, H. T. B. M. et al. Performance of dry-separation processes in the recovery of cenospheres from fly Ash and their implementation in a recovery unit. *Int. J. Min. Process.* **98**, 15–23 (2011).

[101] Vassilev, S. V. et al. Phase-mineral and chemical composition of fractions separated from composite fly ashes at the Soma power station, Turkey. *Int. J. Coal Geol.* **61**, 65–85 (2005).

[102] Dong, Y., Jow, J., Su, J. & Lai, S. Fly ash separation technology and its potential applications. *World of Coal Ash (WOCA) Conference* 22, 25 (2013).

[103] Sharonova, O. M., Natalia, A., Oreshkina, L., Kurteeva, I. & Alexander, G. Aerodynamic separation of fly ashes of selective sampling from pulverized combustion of coals of different ranks. *Eng. Technol.* **5**, 72–86 (2012).

[104] Muratov, B. et al. Physico-Chemical study of the possibility of utilization of coal Ash by processing as secondary Raw materials to obtain a composite cement clinker. *J. Compos. Sci.* **7**, 234 (2023).

[105] Ibrahim, M., Rahman, M. K., Najamuddin, S. K., Alhelal, Z. S. & Acero, C. E. A review on utilization of industrial by-products in the production of controlled low strength materials and factors influencing the properties. *Constr. Build. Mater.* **325**, 126704 (2022).

[106] Sakir, S., Raman, S. N., Safiuddin, M., Kaish, A. B. M. A. & Mutalib, A. A. Utilization of By-Products and wastes as supplementary cementitious materials in structural mortar for sustainable construction. *Sustainability* **12**, 3888 (2020).

[107] Azad, N. M. & Samarakoon, S. M. S. M. K. Utilization of industrial By-Products/Waste to manufacture geopolymer Cement/Concrete. *Sustainability* **13**, 873 (2021).

[108] Kurniati, E. O., Pederson, F. & Kim, H. J. Application of steel slags, ferronickel slags, and copper mining waste as construction materials: A review. *Resour. Conserv. Recycl*. **198**, 107175 (2023).

[109] Taha, Y. et al. Towards an integrated approach for zero coal mine waste storage: solutions based on materials circularity and sustainable resource governance. *Miner. Process. Extr. Metall. Rev.* **44**, 375–388 (2023).

[110] Acordi, J. et al. Waste valorization of coal mining waste from a circular economy perspective: A Brazilian case study based on environmental and physicochemical features. *Resour. Policy*. **80**, 103243 (2023).

[111] ROSIEK, J. The implementation of circular economy concept in the Polish coal combustion products Sector – selected problems. *Economic Environ. Stud.* **18**, 353–373 (2018).

[112] Pactwa, K., Woźniak, J. & Dudek, M. Coal mining waste in Poland in reference to circular economy principles. *Fuel* **270**, 117493 (2020).

[113] Blissett, R. S. & Rowson, N. A. A review of the multi-component utilisation of coal fly Ash. *Fuel* **97**, 1–23 (2012).

[114] Kumar, S., Kristály, F. & Mucsi, G. Geopolymerisation behaviour of size fractioned fly Ash. *Adv. Powder Technol.* **26**, 24–30 (2015).

[115] Zhu, Z., Wang, X., Dai, S., Huang, B. & He, Q. Fractional characteristics of coal fly Ash for beneficial use. *J. Mater. Civ. Eng.* **25**, 63–69 (2013).

[116] Wrona, J., Żukowski, W., Bradło, D. & Czupryński, P. Recovery of cenospheres and fine fraction from coal fly Ash by a novel dry separation method. *Energies (Basel)*. **13**, 3576 (2020).

[117] Zimar, Z. et al. Application of coal fly Ash in pavement subgrade stabilisation: A review. *J. Environ. Manage.* **312**, 114926 (2022). 35364515 10.1016/j.jenvman.2022.114926

[118] Kouakou, C. H. & Morel, J. C. Strength and elasto-plastic properties of non-industrial Building materials manufactured with clay as a natural binder. *Appl. Clay Sci.* **44**, 27–34 (2009).

[119] Le Saoût, G., Lothenbach, B., Hori, A., Higuchi, T. & Winnefeld, F. Hydration of Portland cement with additions of calcium sulfoaluminates. *Cem. Concr Res.* **43**, 81–94 (2013).

[120] Koukouzas, N., Hämäläinen, J., Papanikolaou, D., Tourunen, A. & Jäntti, T. Mineralogical and elemental composition of fly Ash from pilot scale fluidised bed combustion of lignite, bituminous coal, wood chips and their blends. *Fuel* **86**, 2186–2193 (2007).

[121] GOODARZI, F. Characteristics and composition of fly Ash from Canadian coal-fired power plants. *Fuel* **85**, 1418–1427 (2006).

[122] Wang, X. Y. & Park, K. B. Analysis of compressive strength development of concrete containing high volume fly Ash. *Constr. Build. Mater.* **98**, 810–819 (2015).

[123] Delihowski, J. et al. Size fraction characterisation of highly-calcareous and siliceous fly ashes. *J. Therm. Anal. Calorim.* **149**, 10587–10603 (2024).

[124] Delihowski, J., Gajek, M., Izak, P. & Jarosz, M. Thermal studies of fractionated lignite and brown coal fly ashes. *Materials* **17**, 3464 (2024). 39063755 10.3390/ma17143464 PMC11277734

[125] Anastasopoulou, A. Industrial separation processes: Fundamentals. *Green Process. Synthesis* **2** (5), 533–534 (2013).

[126] Hirajima, T. et al. Recovery of cenospheres from coal fly Ash using a dry separation process: separation Estimation and potential application. *Int. J. Min. Process.* **95**, 18–24 (2010).

[127] Gupta, A. Yan. D. Mineral Processing Design and Operations (eds Gupta, A. & Yan, D.). Elsevier, 2nd edn (2016). 10.1016/C2014-0-01236-1

[128] Gray, M. L. et al. Physical cleaning of high carbon fly Ash. *Fuel Process. Technol.* **76**, 11–21 (2002).

[129] Itskos, G., Itskos, S. & Koukouzas, N. Size fraction characterization of highly-calcareous fly Ash. *Fuel Process. Technol.* **91**, 1558–1563 (2010).

[130] Lanzerstorfer, C. Fly Ash from coal combustion: dependence of the concentration of various elements on the particle size. *Fuel* **228**, 263–271 (2018).

[131] Kumar, S., Singh, J. & Mohapatra, S. K. Role of particle size in assessment of physico-chemical properties and trace elements of Indian fly Ash. *Waste Manage. Research: J. Sustainable Circular Econ.* **36**, 1016–1022 (2018). 10.1177/0734242X18804033 30307833

[132] Ryszard, W. Potrzeba ochrony Beidelitowych iłów w KWB Bełchatów. *Przegląd Geologiczny* **41** (9), 612–620 (1993).

[133] Ratajczak, T., Hycnar, E. & Bożęcki, P. The beidellite clays from the Bełchatów lignite deposit as a Raw material for constructing waterproofing barriers. *Gospodarka Surowcami Mineralnymi*. **33**, 53–67 (2017).

[134] Hycnar, E. Mucha, J, Wasilewska-Błaszczyk, M. Ratajczak, T. Documentation of accompanying minerals on the example of limestones from the bełchatów lignite deposit (Central Poland), *Conf.: 18th Inter. Multidisc. Sci. Geoconf.* SGEM2018, Albena, Bulgaria, **18** (1.3) (2018).

[135] Pękala, A. The mineral character and Geomechanical properties of the transitional rocks from the Mesozoic-Neogene contact zone in the Bełchatów lignite deposit. *J. Sustainable Min.* **13**, 10–14 (2014).

[136] Mandal, R., Panda, S. K. & Nayak, S. Rheology of concrete: critical Review, recent Advancements, and future prospectives. *Constr. Build. Mater.* **392**, 132007 (2023).

[137] Macosko, C. Rheology: Principles, Measurements, and applications. *Engineering Mater. Sci. Phy.* **1**, 1–350 (1994).

[138] Larson, R. G. & Wei, Y. A review of Thixotropy and its rheological modeling. *J. Rheol (N Y N Y)*. **63**, 477–501 (2019).

[139] *Theory of Viscoelasticity*. Elsevier, (1982). 10.1016/B978-0-12-174252-2.X5001-7

[140] Hyun, K. et al. A review of nonlinear oscillatory shear tests: analysis and application of large amplitude oscillatory shear (LAOS). *Prog Polym. Sci.* **36**, 1697–1753 (2011).

[141] García-Maté, M., Santacruz, I., De la Torre, Á. G., León-Reina, L. & Aranda, M. A. G. Rheological and hydration characterization of calcium sulfoaluminate cement pastes. *Cem. Concr Compos.* **34**, 684–691 (2012).

[142] Yan, Z., Zhang, H. & Zhu, Y. Hydration kinetics of sulfoaluminate cement with different water/cement ratios as grouting material used for coal mines. *Magazine Concrete Res.* **74**, 1056–1064 (2022).

[143] Mondal, S. K., Clinton, C., Ma, H., Kumar, A. & Okoronkwo, M. U. Effect of class C and class F fly Ash on Early-Age and Mature-Age properties of calcium sulfoaluminate cement paste. *Sustainability* **15**, 2501 (2023).

[144] Fernández-Carrasco, L. & Vázquez, E. Reactions of fly Ash with calcium aluminate cement and calcium sulphate. *Fuel* **88**, 1533–1538 (2009).

[145] Porbaha, A., Pradhan, T. B. S. & Yamane, N. Time effect on shear strength and permeability of fly Ash. *J. Energy Eng.* **126**, 15–31 (2000).

[146] Zhang, W., Wu, F. & Zhang, Y. Early hydration and setting process of fly Ash-blended cement paste under different curing temperatures. *J. Wuhan Univ. Technology-Mater Sci. Ed.* **35**, 551–560 (2020).

[147] Prince, W., Espagne, M. & Aïtcin, P. C. Ettringite formation. *Cem. Concr Res.* **33**, 635–641 (2003).

[148] Famy, C. & Taylor, H. Ettringite in hydration of Portland cement concrete and its occurrence in mature concretes. *ACI Mater. J.* **98**, 350–356 (2001).

[149] John, E. & Lothenbach, B. Cement hydration mechanisms through time – a review. *J. Mater. Sci.* **58**, 9805–9833 (2023).

[150] Ylmén, R., Jäglid, U., Steenari, B. M. & Panas, I. Early hydration and setting of Portland cement monitored by IR, SEM and Vicat techniques. *Cem. Concr Res.* **39**, 433–439 (2009).

[151] Jakob, C. et al. Relating ettringite formation and rheological changes during the initial cement hydration: A comparative study applying XRD Analysis, rheological measurements and modeling. *Materials* **12**, 2957 (2019). 31547266 10.3390/ma12182957 PMC6766233

[152] Malkin, A. Y. & Patlazhan, S. A. Wall slip for complex liquids – Phenomenon and its causes. *Adv. Colloid Interface Sci.* **257**, 42–57 (2018). 29934140 10.1016/j.cis.2018.05.008

[153] Marinho, T. O., Marchesini, F. H., de Oliveira, M. C. K. & Nele, M. Apparent wall slip effects on rheometric measurements of waxy gels. *J. Rheol (N Y N Y)*. **65**, 257–272 (2021).

[154] Tianhong Chen. Preventing wall slip in rheology experiments. *Applications TA Instruments* 1–3 (2004). https://www.tainstruments.com/applications-notes/preventing-wall-slip-in-rheology-experiments/

[155] Priezjev, N. V., Darhuber, A. A. & Troian, S. M. Slip behavior in liquid films on surfaces of patterned wettability: comparison between continuum and molecular dynamics simulations. *Phys. Rev. E*. **71**, 041608 (2005). 10.1103/PhysRevE.71.041608 15903683

[156] Nazar, S., Yang, J., Thomas, B. S., Azim, I. & Ur Rehman, S. K. Rheological properties of cementitious composites with and without nano-materials: A comprehensive review. *J. Clean. Prod.* **272**, 122701 (2020).

[157] Roussel, N., Ovarlez, G., Garrault, S. & Brumaud, C. The origins of Thixotropy of fresh cement pastes. *Cem. Concr Res.* **42**, 148–157 (2012).

[158] Corstanje, W. A., Stein, H. N. & Stevels, J. M. Hydration reactions in pastes C3S + C3A + CaSO4.2aq + H2O at 25°C.I. *Cem. Concr Res.* **3**, 791–806 (1973).

[159] Alterary, S. S. & Marei, N. H. Fly Ash properties, characterization, and applications: A review. *J. King Saud Univ. Sci.* **33**, 101536 (2021).

[160] Iribarne, A. P., Iribarne, J. V. & Anthony, E. J. Reactivity of calcium sulfate from FBC systems. *Fuel* **76**, 321–327 (1997).

[161] Bentz, D. P., Ferraris, C. F., Galler, M. A., Hansen, A. S. & Guynn, J. M. Influence of particle size distributions on yield stress and viscosity of cement–fly Ash pastes. *Cem. Concr Res.* **42**, 404–409 (2012).

[162] Luce, R. W., Bartlett, R. W. & Parks, G. A. Dissolution kinetics of magnesium silicates. *Geochim. Cosmochim. Acta*. **36**, 35–50 (1972).

[163] Nishiki, Y. et al. Formation of magnesium silicate hydrate (M-S-H) at pH 10 and 50°C in open-flow systems. *Appl. Geochem.* **148**, 105544 (2023).

[164] Gardner, L. J. et al. Characterisation of magnesium potassium phosphate cements blended with fly Ash and ground granulated blast furnace slag. *Cem. Concr Res.* **74**, 78–87 (2015).

[165] Boutkhil, H. et al. Strength characteristics and rheological behavior of a high level of fly Ash in the production of concrete. *ACS Omega*. **9**, 14419–14428 (2024). 38559963 10.1021/acsomega.4c00147 PMC10976435

[166] Alrefaei, Y., Wang, Y. S., Qian, Y. & Dai, J. G. Effects of solid activator and fly Ash on rheology and Thixotropy of One-Part Alkali-Activated pastes. *J. Adv. Concr. Technol.* **20**, 139–151 (2022).

[167] Hower, J. C. et al. Coal-derived unburned carbons in fly ash: A review. *Int. J. Coal Geol.* **179**, 11–27 (2017).

[168] Wu, C. R., Tang, W., Huo, Y. L., Zhan, B. J. & Kou, S. C. Investigation of fresh properties of Self-Leveling Cement-Based pastes with CFB fly Ash as an SCM. *Buildings* **15**, 966 (2025).

[169] Pelletier-Chaignat, L. et al. Influence of the calcium sulphate source on the hydration mechanism of Portland cement–calcium sulphoaluminate clinker–calcium sulphate binders. *Cem. Concr Compos.* **33**, 551–561 (2011).

[170] Álvarez-Pinazo, G., Santacruz, I. & Aranda, M. A. G. De La Torre, Á. G. Hydration of belite–ye’elimite–ferrite cements with different calcium sulfate sources. *Adv. Cem. Res.* **28**, 529–543 (2016).

[171] Zhang, L. & Glasser, F. P. Hydration of calcium sulfoaluminate cement at less than 24 h. *Adv. Cem. Res.* **14**, 141–155 (2002).

[172] Zhang, J., Ke, G. & Liu, Y. Early hydration heat of calcium sulfoaluminate cement with influences of supplementary cementitious materials and water to binder ratio. *Materials* **14**, 642 (2021). 33573301 10.3390/ma14030642 PMC7866813

[173] Daniels, K. A. et al. Gel formation at the front of expanding calcium bentonites. *Minerals* **11**, 215 (2021).

[174] Dananaj, I., Frankovská, J. & Janotka, I. The influence of smectite content on microstructure and geotechnical properties of calcium and sodium bentonites. *Appl. Clay Sci.* **28**, 223–232 (2005).

[175] Ines García-Lodeiro. Hybrid alkaline cements. Part I: fundamentals. *Romanian J. Mater.* **42**, 330–335 (2012).

[176] Xiao, B., Miao, S., Gao, Q., Chen, B. & Li, S. Hydration mechanism of sustainable Clinker-Free steel slag binder and its application in mine backfill. *JOM* **73**, 1053–1061 (2021).

[177] Nath, S. K. & Kumar, S. Role of particle fineness on engineering properties and microstructure of fly Ash derived geopolymer. *Constr. Build. Mater.* **233**, 117294 (2020).

[178] Zhuang, X. Y. et al. Fly ash-based geopolymer: clean production, properties and applications. *J. Clean. Prod.* **125**, 253–267 (2016).

[179] Vogt, U. Ballschmiede & Koenders. Reactivity and microstructure of Metakaolin based geopolymers: effect of fly Ash and Liquid/Solid contents. *Materials* **12**, 3485 (2019). 31653060 10.3390/ma12213485 PMC6862292

[180] Temuujin, J., van Riessen, A. & Williams, R. Influence of calcium compounds on the mechanical properties of fly Ash geopolymer pastes. *J. Hazard. Mater.* **167**, 82–88 (2009). 19201089 10.1016/j.jhazmat.2008.12.121

[181] Neaman, A. & Singer, A. Rheological properties of aqueous suspensions of Palygorskite. *Soil Sci. Soc. Am. J.* **64**, 427–436 (2000).

[182] Yahia, A. Effect of solid concentration and shear rate on shear-thickening response of high-performance cement suspensions. *Constr. Build. Mater.* **53**, 517–521 (2014).

[183] Ran, R. et al. Understanding the rheology of kaolinite clay suspensions using bayesian inference. *J. Rheol (N Y N Y)*. **67**, 241–252 (2023).

[184] Bingham, E. C. Eugene Cook. Fluidity and plasticity. McGraw-Hill, *McGraw-Hill* (1922). (1922).

[185] Fischer, P. Rheometry of Pastes, suspensions and granular Materials – Application in industry and environment. *Appl. Rheology*. **16**, 181–181 (2006).

[186] Vance, K., Kumar, A., Sant, G. & Neithalath, N. The rheological properties of ternary binders containing Portland cement, limestone, and Metakaolin or fly Ash. *Cem. Concr Res.* **52**, 196–207 (2013).

[187] Wójcik, Ł., Izak, P. & Kuś, R. The influence of composition changes on properties of clay-cement binders. *Ceramika Materiały Ogniotrwałe ISSN*. **-1505-1269 61**, 27–30 (2009).

[188] Izak, P., Wójcik, Ł. & Słowikowski, D. Rheology of soil binder dispersions. *Ceramika Materiały Ogniotrwałe; ISSN: 1505 – 1269*. **67**, 162–163 (2015).

[189] Luckham, P. F. & Rossi, S. The colloidal and rheological properties of bentonite suspensions. *Adv. Colloid Interface Sci.* **82**, 43–92 (1999).

[190] Mostafa, A. M. & Yahia, A. Physico-chemical kinetics of structural build-up of neat cement-based suspensions. *Cem. Concr Res.* **97**, 11–27 (2017).

[191] Ovarlez, G., Tocquer, L., Bertrand, F. & Coussot, P. Rheopexy and tunable yield stress of carbon black suspensions. *Soft Matter*. **9**, 5540 (2013).

[192] Neuville, M. et al. Rheology of a gypsum suspension in the presence of different superplasticizers. *J. Rheol (N Y N Y)*. **56**, 435–451 (2012).

[193] Demeusy, Y., Gauffinet, S. & Labbez, C. Paste Rheology and Surface Charge of Calcined Kaolinite. (2023).

[194] Fu, X., Liu, Y., Lu, J. & Sun, R. Order-disorder transition during shear thickening in bidisperse dense suspensions. *J. Colloid Interface Sci.* **662**, 1044–1051 (2024). 38387366 10.1016/j.jcis.2024.02.033

[195] Catherall, A. A., Melrose, J. R. & Ball, R. C. Shear thickening and order–disorder effects in concentrated colloids at high shear rates. *J. Rheol (N Y N Y)*. **44**, 1–25 (2000).

[196] Gurnon, A. K. & Wagner, N. J. Microstructure and rheology relationships for shear thickening colloidal dispersions. *J. Fluid Mech.* **769**, 242–276 (2015).

[197] Kessler, V. G. Sol–Gel precursors. in The Sol-Gel Handbook 195–224 (Wiley, doi:10.1002/9783527670819.ch06. (2015).

[198] Koohestani, B., Mokhtari, P., Yilmaz, E., Mahdipour, F. & Darban, A. K. Geopolymerization mechanism of binder-free mine tailings by sodium silicate. *Constr. Build. Mater.* **268**, 121217 (2021).

[199] Fernández-Jiménez, A., Vázquez, T. & Palomo, A. Effect of sodium silicate on calcium aluminate cement hydration in highly alkaline media: A microstructural characterization. *J. Am. Ceram. Soc.* **94**, 1297–1303 (2011).

[200] Robayo, R. A., Mulford, A. & Munera, J. Mejía de Gutiérrez, R. Alternative cements based on alkali-activated red clay brick waste. *Constr. Build. Mater.* **128**, 163–169 (2016).

[201] Guo, S. et al. Delaying the hydration of Portland cement by sodium silicate: setting time and retarding mechanism. *Constr. Build. Mater.* **205**, 543–548 (2019).

[202] Wang, Z., Sun, Y., Zhang, S. & Wang, Y. Effect of sodium silicate on Portland cement/calcium aluminate cement/gypsum rich-water system: strength and microstructure. *RSC Adv.* **9**, 9993–10003 (2019). 35558996 10.1039/c8ra09901d PMC9088715

[203] Fernández-Jiménez, A. & Palomo, A. Composition and microstructure of alkali activated fly Ash binder: effect of the activator. *Cem. Concr Res.* **35**, 1984–1992 (2005).

[204] Vance, K., Dakhane, A., Sant, G. & Neithalath, N. Observations on the rheological response of alkali activated fly Ash suspensions: the role of activator type and concentration. *Rheol Acta*. **53**, 843–855 (2014).

[205] Krizan, D. & Zivanovic, B. Effects of dosage and modulus of water glass on early hydration of alkali–slag cements. *Cem. Concr Res.* **32**, 1181–1188 (2002).

[206] Roussel, N. Rheology of fresh concrete: from measurements to predictions of casting processes. *Mater. Struct.* **40**, 1001–1012 (2007).

[207] Dullaert, K. & Mewis, J. A structural kinetics model for Thixotropy. *J. Nonnewton Fluid Mech.* **139**, 21–30 (2006).

[208] Barnes, H. A. Thixotropy—a review. *J. Nonnewton Fluid Mech.* **70**, 1–33 (1997).

[209] Dullaert, K., Mewis, J. & Thixotropy Build-up and breakdown curves during flow. *J. Rheol (N Y N Y)*. **49**, 1213–1230 (2005).

[210] Shoaib, M., Cruz, N. & Bobicki, E. R. Effect of pH-modifiers on the rheological behaviour of clay slurries: difference between a swelling and non-swelling clay. *Colloids Surf. Physicochem Eng. Asp*. **643**, 128699 (2022).

[211] Landrou, G., Brumaud, C. & Habert, G. Clay particles as binder for Earth buildings materials: a fresh look into rheology of dense clay suspensions. *EPJ Web Conf.* **140**, 13010 (2017).

[212] Du, M., Liu, J., Clode, P. & Leong, Y. K. Microstructure and rheology of bentonite slurries containing multiple-charge phosphate-based additives. *Appl. Clay Sci.* **169**, 120–128 (2019).

[213] Cui, Q., Maierdan, Y., Chen, B., Ge, J. & Liu, N. Comparative research on the application of slag as an alternative to cement in binder-bentonite cutoff wall backfills. *Constr. Build. Mater.* **325**, 126817 (2022).

[214] Duong, L. H. & Nguyen, M. N. Colloidal interaction of fly Ash and soil clay. *Colloids Surf. Physicochem Eng. Asp*. **692**, 133944 (2024).

[215] Erdoğan, S. T., Martys, N. S., Ferraris, C. F. & Fowler, D. W. Influence of the shape and roughness of inclusions on the rheological properties of a cementitious suspension. *Cem. Concr Compos.* **30**, 393–402 (2008).

[216] Kuenzel, C. & Ranjbar, N. Dissolution mechanism of fly Ash to quantify the reactive aluminosilicates in geopolymerisation. *Resour. Conserv. Recycl*. **150**, 104421 (2019).

[217] El Fami, N., Ez-zaki, H., Sassi, O., Boukhari, A. & Diouri, A. Rheology, calorimetry and electrical conductivity related-properties for monitoring the dissolution and precipitation process of cement-fly Ash mixtures. *Powder Technol.* **411**, 117937 (2022).

[218] Kolawole, J. T., Combrinck, R. & Boshoff, W. P. Rheo-viscoelastic behaviour of fresh cement-based materials: cement paste, mortar and concrete. *Constr. Build. Mater.* **248**, 118667 (2020).

[219] Gonet, A., Stryczek, S. & Kremieniewski, M. Modern methods of strengthening and sealing salt mines. *Energies (Basel)*. **15**, 5303 (2022).

[220] Basga, S. D., Tsozue, D., Temga, J. P., Balna, J. & Nguetnkam, J. P. Land use impact on clay dispersion/flocculation in irrigated and flooded vertisols from Northern Cameroon. *Int. Soil. Water Conserv. Res.* **6**, 237–244 (2018).

[221] Kameda, J. & Morisaki, T. Sensitivity of clay suspension rheological properties to pH, Temperature, Salinity, and Smectite-Quartz ratio. *Geophys. Res. Lett.* **44**, 9615–9621 (2017).

[222] Kwan, A. K. H. & Chen, J. J. Adding fly Ash microsphere to improve packing density, flowability and strength of cement paste. *Powder Technol.* **234**, 19–25 (2013).

[223] Evans, J. C. & Larrahondo, J. M. Yeboah, N. N. N. Fate of bentonite in slag–cement–bentonite slurry trench cut-off walls for polluted sites. *Environ. Geotechnics*. **10**, 319–331 (2023).

[224] Cao, B., Zhang, Y. & Al-Tabbaa, A. SEBS-Polymer-Modified Slag–Cement–Bentonite for resilient slurry walls. *Sustainability* **14**, 2093 (2022).

[225] Ma, J., Zhang, H., Wang, D., Wang, H. & Chen, G. Rheological properties of cement paste containing ground fly Ash based on particle morphology analysis. *Cryst. (Basel)*. **12**, 524 (2022).

[226] Wu, H. L., Jin, F., Ni, J. & Du, Y. J. Engineering properties of vertical cutoff walls consisting of reactive Magnesia-Activated slag and bentonite: Workability, Strength, and hydraulic conductivity. *J. Mater. Civil Eng.* **31** (11), 04019263 (2019).

[227] Szostak, B. & Golewski, G. L. Rheology of cement pastes with siliceous fly Ash and the CSH Nano-Admixture. *Materials* **14**, 3640 (2021). 34209995 10.3390/ma14133640 PMC8269633

[228] Schultz, M. A. & Struble, L. J. Use of oscillatory shear to study flow behavior of fresh cement paste. *Cem. Concr Res.* **23**, 273–282 (1993).

[229] Huang, T. et al. Evaluation of microstructural changes in fresh cement paste using AC impedance spectroscopy vs. oscillation rheology and 1H NMR relaxometry. *Cem. Concr Res.* **149**, 106556 (2021).

[230] Assaad, J. Khayat, K. Effect of viscosity-enhancing admixtures on formwork pressure and thixotropy of self-consolidating concrete. *ACI Mater. J.* **103**, 280–287 (2006).

[231] Zhang, K., Mezhov, A. & Schmidt, W. Chemical and thixotropic contribution to the structural build-up of cementitious materials. *Constr. Build. Mater.* **345**, 128307 (2022).

[232] Cao, B., Chen, J. & Al-Tabbaa, A. Crack-resistant cement–bentonite cut-off wall materials incorporating superabsorbent polymers. *Can. Geotech. J.* **58**, 800–810 (2021).

